# A perioperative surgeon-controlled open-lung approach versus conventional protective ventilation with low positive end-expiratory pressure in cardiac surgery with cardiopulmonary bypass (PROVECS): study protocol for a randomized controlled trial

**DOI:** 10.1186/s13063-018-2967-y

**Published:** 2018-11-13

**Authors:** David Lagier, François Fischer, William Fornier, Jean-Luc Fellahi, Pascal Colson, Bernard Cholley, Samir Jaber, Karine Baumstarck, Catherine Guidon, for the PROVECS investigators and the ARCOTHOVA group, David Lagier, Gabrielle Quintana, Françoise Gaillat, Patrice Nedir, Raphaelle Duponq, Romain Gomert, Benoit Guinard, Florent Heraud, Catherine Guidon, Judith Villacorta, Su Degirmenci, Nicolas Pernoud, Pascal Colson, Gianluca Samarani, Marion Lalande, Jean-Luc Fellahi, William Fornier, Bernard Cholley, Thi Mum Huynh, François Fischer, Cecile Gros, Faycal Elmiloudi, Charles Tacquard, Audrey Bilger, François Levy, Ecaterina Cinca, Clément Bongarzone, Bob Heger, Victor Balvay, Marjory Berns, Walid Oulehri, Alexandre Ouattara

**Affiliations:** 1 Department of Cardiovascular Anesthesiology and Critical Care Medicine, La Timone University Hospital, AP-HM and Aix-Marseille University, 264 rue saint Pierre, 13005, cedex 5, Marseille, France; 2 Department of Cardiovascular and Thoracic Anesthesiology, Nouvel Hôpital Civil, Strasbourg, France; 3 Department of Anesthesiology and Critical Care Medicine, Louis Pradel University Hospital and University Claude Bernard, 28 Avenue du Doyen Lépine, 69677 Bron, France; 4 Department of Anesthesiology and Critical Care Medicine, Arnaud de Villeneuve University Hospital, 371 Avenue du Doyen Gaston Giraud, 34295 Montpellier, France; 5 Department of Anesthesiology and Critical Care Medicine, Hôpital Européen Georges Pompidou, AP-HP and University Paris Descartes-Sorbonne Paris Cité, 20 Rue Leblanc, 75015 Paris, France; 6 Department of Anesthesiology and Critical Care Medicine, Saint Eloi University Hospital, 80 Avenue Augustin Fliche, 34295 Montpellier, France; 7 Unité de Recherche EA3279, Aix-Marseille University, 27 bd Jean Moulin, Marseille, cedex 5, 13385 Marseille, France; 8 Anesthésie Réanimation Cœur Thorax Vaisseaux, SAR II – Hôpital Haut-Lévèque, Avenue Magellan, 33600 Pessac, France

**Keywords:** Postoperative pulmonary complications, Cardiac surgery, Cardiopulmonary bypass, Mechanical ventilation, Positive end-expiratory pressure

## Abstract

**Background:**

Postoperative pulmonary complications (PPCs) are frequent after on-pump cardiac surgery. Cardiac surgery results in a complex pulmonary insult leading to high susceptibility to perioperative pulmonary atelectasis. For technical reasons, ventilator settings interact with the surgical procedure and traditionally, low levels of positive end-expiratory pressure (PEEP) have been used. The objective is to compare a perioperative, multimodal and surgeon-controlled open-lung approach with conventional protective ventilation with low PEEP to prevent PPCs in patients undergoing cardiac surgery.

**Methods/design:**

The perioperative open-lung protective ventilation in cardiac surgery (PROVECS) trial is a multicenter, two-arm, randomized controlled trial. In total, 494 patients scheduled for elective cardiac surgery with cardiopulmonary bypass (CPB) and aortic cross-clamp will be randomized into one of the two treatment arms. In the experimental group, systematic recruitment maneuvers and perioperative high PEEP (8 cmH2O) are associated with ultra-protective ventilation during CPB. In this group, the settings of the ventilator are controlled by surgeons in relation to standardized protocol deviations. In the control group, no recruitment maneuvers, low levels of PEEP (2 cmH2O) and continuous positive airway pressure during CPB (2 cmH2O) are used. Low tidal volumes (6–8 mL/kg of predicted body weight) are used before and after CPB in each group. The primary endpoint is a composite of the single PPCs evaluated during the first 7 postoperative days.

**Discussion:**

The PROVECS trial will be the first multicenter randomized controlled trial to evaluate the impact of a perioperative and multimodal open-lung ventilatory strategy on the occurrence of PPCs after on-pump cardiac surgery. The trial design includes standardized surgeon-controlled protocol deviations that guarantee a pragmatic approach. The results will help anesthesiologists and surgeons aiming to optimize ventilatory settings during cardiac surgery.

**Trial registration:**

Clinical Trials.gov, NCT 02866578. Registered on 15 August 2016. Last updated 11 July 2017.

**Electronic supplementary material:**

The online version of this article (10.1186/s13063-018-2967-y) contains supplementary material, which is available to authorized users.

## Background

Postoperative pulmonary complications (PPCs) remain a frequent event after on-pump cardiac surgery [1]. PPCs are responsible for significant morbidity and mortality [2]. They are mostly characterized by transient hypoxemia (up to 25%) while acute respiratory distress syndrome and postoperative pneumonia are less frequently encountered [3]. The use of high-flow nasal oxygen therapy and non-invasive ventilation is necessary to treat the most severe forms of respiratory failure [4], leading to prolonged stays in both the intensive care unit (ICU) and in hospital in general.

General anesthesia with invasive mechanical ventilation induces its own lung insult, which has been widely described as ventilator-induced lung injury [5]. A second pulmonary hit is more specific to cardiac surgery. Cardiopulmonary bypass (CPB) effectively activates a systemic inflammatory response [6] and the aortic cross clamp is responsible for lung ischemic injury [7]. Moreover, complete sternotomy, frequent blood transfusions and postoperative pain are involved in the high incidence of PPCs [8–10]. At the pulmonary level, cardiac surgery is related to increased permeability of the alveolo-capillary barrier [11, 12] and mucociliary dysfunction [13]. Pulmonary atelectasis is very common in this context, [14, 15].

Preventing PPCs with specific perioperative ventilatory management is not a new approach [16]. Many reports have described the concept of protective ventilation [17, 18]. Inspired by the results obtained in critical care medicine in patients with acute respiratory distress syndrome (ARDS) [19], the use of low tidal volumes (6–8 mL/kg predicted body weight) has spread to the operating theater [20–22] and there is now an established consensus [23]. However, the use of low tidal volumes may precipitate the constitution of pulmonary atelectasis in the poorly ventilated, dependent regions of the lung [16]. The open-lung approach corresponds to the use of systematic recruitment maneuvers (“open the lung”) associated with high levels of end-expiratory pressure (“keep it open”) in order to prevent atelectasis [24]. The efficacy of the open-lung approach in preventing atelectasis has been well-described in cardiac surgery preclinical studies [25, 26]. Nonetheless, the clinical effectiveness of open-lung ventilation during general anesthesia [23] or in patients with ARDS [27] is still a matter of debate. The largest randomized trials evaluating the open-lung approach during abdominal surgery [28, 29] have not found any benefit in using recruitment maneuvers and higher positive end-expiratory pressure (PEEP). The theoretical interest of preventing pulmonary atelectasis during the mechanical ventilation phase could be lost after tracheal extubation, when PPCs appear. Moreover, the hemodynamic safety of high ventilatory pressure has been questioned [29, 30].

In cardiac surgery, high levels of PEEP have not historically been used because of the technical interference induced by the movements of the lung in the operative field, particularly in the case of a pleural opening. Moreover, the hemodynamic consequences may be more severe in patients undergoing cardiac surgery. During CPB, lung ventilation is still widely interrupted because of the absence of lung perfusion and for surgical comfort [31]. Maintaining lung ventilation with or without perfusion has shown positive effects on the inflammatory response [32] and on post-CPB gas exchange [33, 34]. However, this effect seems to be short term and there is no sufficient clinical evidence to support specific ventilatory management during CPB [35]. Regarding the high incidence of pulmonary atelectasis in cardiac surgery, the benefit of a multimodal and perioperative open-lung approach, including lung ventilation during CPB, has been suggested [36]. However, because of the potential impact on the surgical procedure and cardiac function, the use of open-lung ventilation in cardiac surgery needs to be justified by the highest level of clinical evidence. We hypothesize that using systematic recruitment maneuvers, higher PEEP and ventilation during CPB will prevent PPCs after cardiac surgery. We will compare a perioperative open-lung approach involving surgeon-controlled maximization of alveolar recruitment with the conventional low-PEEP strategy.

The primary objective is to assess the efficacy of the perioperative open-lung strategy in terms of PPC incidence; the secondary objectives are to assess the use of specific ventilatory support, postoperative extra pulmonary complications, adverse events and the number of ICU-free days by postoperative day 7.

## Methods/design

### Design

**Fig. 1:**
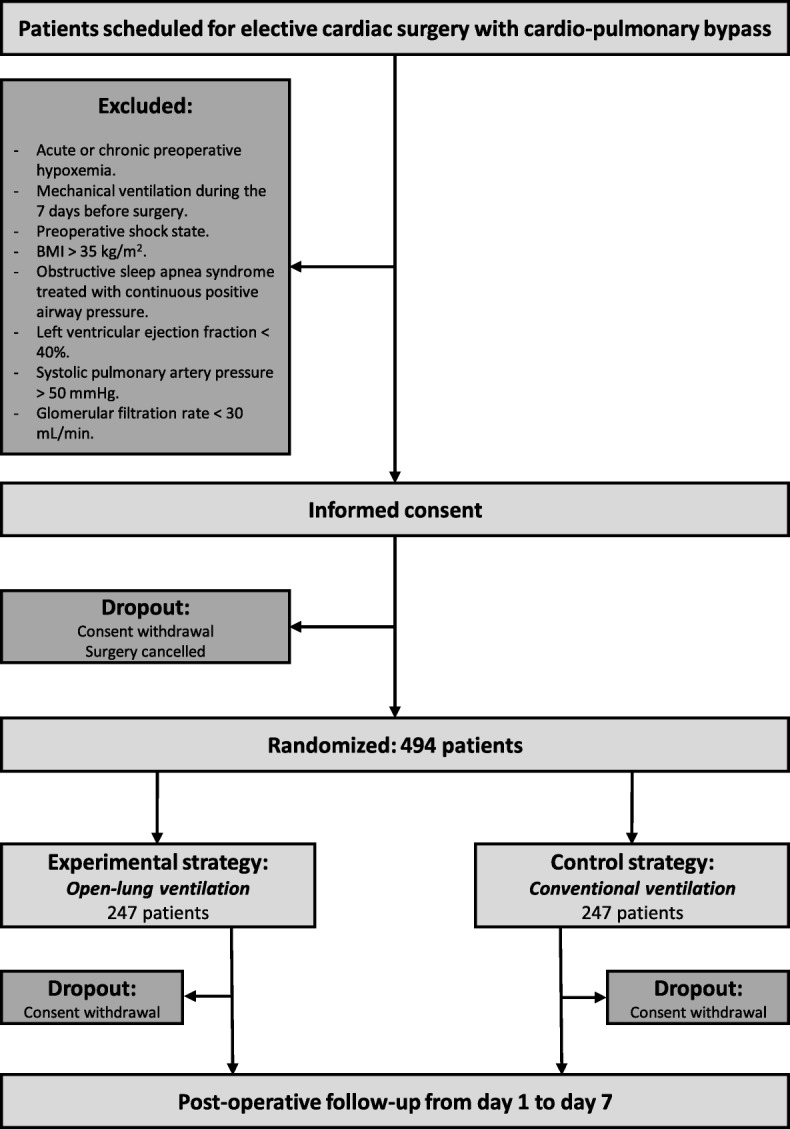
Consolidated Standards of Reporting Trials (CONSORT) diagram for the PROVECS trial. BMI body mass index

PROVECS is a prospective, multicenter, randomized, controlled, two-arm trial comparing two perioperative ventilatory strategies in cardiac surgery with cardiopulmonary bypass: (1) experimental strategy: surgeon-controlled open-lung ventilation; (2) control strategy: conventional protective ventilation with low PEEP (Fig. 1). Double-blinding is ensured by the general anesthesia in the trial participants, and by masking the outcome assessor. Hiding all the intraoperative data (including ventilator settings) on the electronic case report form (CRF) at the end of surgery ensures the masking of the treatment arm Additional file 1. A checklist of recommended items to address in a clinical trial protocol according to the "Standard Protocol Items: Recommendations for Interventional Trials (SPIRIT) 2013 is provided in Additional file 2.

### Partners

The patients will be recruited in six French adult cardiac surgery departments. The methodological support will be provided by the Clinical Research Unit (Unité Aide Méthodologique à la Recherche Clinique, Assistance Publique – Hôpitaux de Marseille, France). The study is sponsored by the Assistance Publique des Hôpitaux de Marseille (Project Manager, Patrick Sudour). This work is supported by institutional grants from the French Clinical Research Program 2015 (Programme Hospitalier de Recherche Clinique). All the details are provided in Table 1.

**Table 1:** PROVECS investigators

Site number	Inclusion center	Investigator(s)	Email address(es)
001	Department of Anesthesiology and Intensive Care Medicine 2, University Hospital La Timone, Assistance Publique Hôpitaux de Marseille	Lagier, David Quintana, Gabrielle Gaillat, Françoise Nedir, Patrice Duponq, Raphaelle Gomert, Romain Guinard, Benoit Heraud, Florent Guidon, Catherine Villacorta, Judith Degirmenci, Su Pernoud, Nicolas	david.lagier@ap-hm.fr gabrielle.quintana@ap-hm.fr francoise.gaillat@ap-hm.fr patricechristian.nedir@ap-hm.fr raphaele.duponq@ap-hm.fr romain.gomert@ap-hm.fr benoit.guinard@ap-hm.fr florent.heraud@ap-hm.fr catherine.guidon@ap-hm.fr judith.villacortatorres@ap-hm.fr su-emmanuelle.degirmenci@ap-hm.fr nicolas.pernoud@ap-hm.fr
002	Department of Anesthesiology and Intensive Care Medicine D, Arnaud de Villeneuve University Hospital, Montpellier	Colson, Pascal Samarani, Gianluca Lalande, Marion	p-colson@chu-montpellier.fr g-samarani@chu-montpellier.fr lalande.marion@gmail.com
003	Department of Anesthesiology and Intensive Care Medicine, University Hospital Louis Pradel – Hospices Civils de Lyon	Fellahi, Jean-Luc Fornier, William	jean-luc.fellahi@chu-lyon.fr w_fornier@yahoo.fr
004	Department of Anesthesiology and Intensive Care Medicine, Hôpital Européen Georges Pompidou, Assistance Publique des Hôpitaux de Paris	Cholley, Bernard Huynh, Thi Mum	bernard.cholley@aphp.fr thimum@free.fr
005	Department of Anesthesiology and Intensive Care Medicine, Nouvel Hôpital Civil, University Hospital of Strasbourg	Fischer, François Gros, Cecile Elmiloudi, Faycal Tacquard, Charles Bilger, Audrey Levy, François Cinca, Ecaterina Bongarzone, Clément Heger, Bob Balvay, Victor Berns, Marjory Oulehri, Walid	francois.fischer1@chru-strasbourg.fr cecile.gros@chru-strasbourg.fr faycal.elmiloudi@chru-strasbourg.fr charlesambroise.tacquard@chru-strasbourg.fr audrey.bilger@chru-strasbourg.fr francois.levy@chru-strasbourg.fr ecaterina.cinca@chru-strasbourg.fr clement.bongarzone@chru-strasbourg.fr bob.heger@chru-strasbourg.fr victor-edouard.balvay@chru-strasbourg.fr marjory.berns@chru-strasbourg.fr walid.oulehri@chru-strasbourg.fr
006	Department of Anesthesiology and Intensive Care Medicine, Service d’Anesthésie-Réanimation SUD Centre Médico-Chirurgical Magellan, Pessac, University Hospital of Bordeaux	Ouattara, Alexandre	alexandre.ouattara@chu-bordeaux.fr

### Study population

#### Inclusion criteria

Patients are eligible if they are scheduled for elective cardiac surgery with general anesthesia, invasive mechanical ventilation, conventional CPB, aortic cross clamp and complete median sternotomy. All patients will be included after providing written, signed, informed consent. Eligible surgeons are defined as cardiac surgery physicians licensed for at least 2 years, working in high-volume university hospital centers with a minimum of 400 surgical operations with CPB each year.

#### Exclusion criteria

The exclusion criteria are surgery or patient related. The surgery-related criteria are:

1. Emergent surgery including cardiac transplantation, aortic dissection and active endocarditis surgery
2. Left ventricular assist device implantation
3. Surgery with circulatory arrest
4. Redo surgery

The patient-related criteria are:

1. Age < 18 years
2. Acute or chronic hypoxemia defined by partial pressure of arterial oxygen (PaO2) < 65 mmHg or pulse oximetry < 95% on ambient air
3. Mechanical ventilation in the 7 days prior to surgery
4. Preoperative shock
5. Body mass index (BMI) > 35 kg/m^2^
6. Obstructive sleep apnea syndrome treated with continuous positive airway pressure
7. Preoperative left ventricular ejection fraction < 40%
8. Right ventricular systolic dysfunction (Doppler-derived tricuspid lateral annular systolic velocity < 10 cm∙s^− 1^)
9. Systolic pulmonary artery pressure > 50 mmHg
10. Glomerular filtration rate < 30 mL∙min^− 1^

### Interventions

Mechanical ventilation is performed with anesthesia and ICU ventilators set on volume-controlled ventilation. All patients are ventilated with low tidal volumes before and after the CPB (6–8 mL/kg of predicted body weight). The predicted body weight is calculated with the formula: 50 + 0.91 × (Height in cm – 152.4) in men and 45.5 + 0.91 × (height in cm – 152.4) in women. The respiratory rate will be freely adjusted before and after CPB by the anesthesiologist to maintain end-tidal CO2 partial pressure between 35 and 45 mmHg. The lowest fraction of inspired oxygen (FiO2) will be targeted in both groups to maintain SpO2 > 94%. The inspiratory to expiratory ratio (I:E) is set at 1:2 (Table 2).

**Table 2:** Perioperative ventilatory protocol in each of the two treatment arms

	Conventional ventilation	Open-lung ventilation
Ventilation before CPB	• Tidal volume 6–8 mL/kg PBW • PEEP 2 cmH2O • RR for ETCO2 35–45 mmHg • Lowest FiO2 to maintain SpO2 > 94%. • I:E ratio at 1:2	• Tidal volume 6–8 mL/kg PBW • PEEP 8 cmH2O • RR for ETCO2 35–45 mmHg • Lowest FiO2 to maintain SpO2 > 94% • I:E ratio at 1:2
Systematic recruitment maneuvers	No	Yes
Ventilation during CPB	CPAP 2 cmH2O	Ultraprotective ventilation • Tidal volume 3 mL/kg PBW • PEEP 8 cmH2O • RR 12 cpm • FiO2 40%
Ventilation after CPB (including in ICU)	• Tidal volume 6–8 mL/kg PBW • PEEP 2 cmH2O • RR for ETCO2 35––45 mmHg • Lowest FiO2 to maintain SpO2 > 94% • I:E ratio at 1:2	• Tidal volume 6–8 mL/kg PBW • PEEP 8 cmH2O • RR for ETCO2 35––45 mmHg • Lowest FiO2 to maintain SpO2 > 94% • I:E ratio at 1:2
Protocol deviation	Rescue strategy • Unplanned recruitment maneuver • +/− PEEP increase	Surgical or hemodynamic deviation • Recruitment maneuver interruption • PEEP decrease (1 cmH2O by 1 cmH2O step)

*CPB* cardiopulmonary bypass, *CPAP* continuous positive airway pressure, *FiO2* inspired oxygen fraction, *I:E* inspiratory time to expiratory time ratio, *PEEP* positive end-expiratory pressure, *PBW* predicted body weight, *RR* respiratory rate, *SpO2* pulse oximetry, *ETCO2* end-tidal CO2

#### Experimental strategy: Surgeon-controlled open-lung ventilation

In the experimental open-lung group, recruitment maneuvers (continuous positive airway pressure maintained at 30 cmH2O for 30 s) are systematically implemented at predefined stages in the surgical procedure:

1. After intubation and invasive arterial line placement
2. After CPB initiation when targeted blood-flow is reached
3. Before aortic de-clamping, after standard balloon de-airing maneuvers
4. At ICU arrival with the ICU ventilator
5. After each breathing circuit disconnection

PEEP levels in the experimental open-lung group are set at 8 cmH2O from intubation in the operating room to extubation in the ICU. During CPB, ultraprotective ventilation is used with PEEP at 8 cmH2O, very low tidal volumes (3 mL/kg of predicted body weight), a respiratory rate of 12 cycles per minute and FiO2 of 40%. Surgical protocol deviation has been standardized (see below and Table 2).

#### Control strategy: conventional protective ventilation with low PEEP

No recruitment maneuvers are carried out. The PEEP is set at 2 cmH2O from intubation to extubation. Continuous positive airway pressure is maintained at 2 cmH2O during CPB (Table 2).

### Protocol deviation

**Fig. 2:**
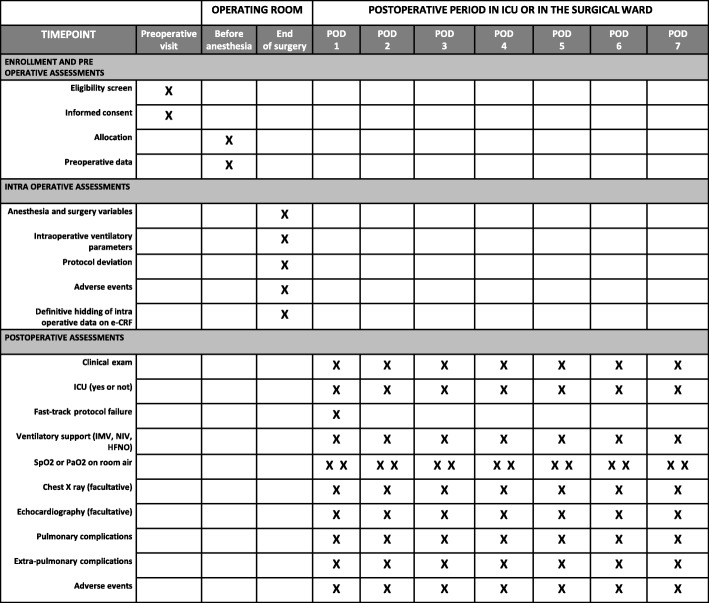
PROVECS trial schedule during the study period. *eCRF* electronic case report form, *HFNO* high-flow nasal oxygen therapy, *ICU* intensive care unit, *IMV* intensive mechanical ventilation, *NIV* non-invasive ventilation, *PaO2* arterial pressure in oxygen, *POD* postoperative day, *SpO2* pulse oximetry

In the experimental strategy group, the recruitment maneuver before and after the CPB can be avoided or interrupted on surgical demand or in the case of systolic arterial pressure < 80 mmHg despite the adequate use of fluids and/or vasoactive drugs. The recruitment maneuver during CPB can be interrupted on surgical demand, or in case of a severe decrease in venous return with the inability to maintain the blood flow. PEEP levels can be decreased on surgical demand or on the anesthesiologist’s decision in the case of hemodynamic impairment despite the adequate use of fluids and/or vasoactive drugs. In these cases, PEEP will be decreased in stages of 1 cmH2O until correction of the problem. In the conventional strategy group, in the case of intraoperative hypoxemia (SpO2 < 92% despite FiO2 80%), unplanned recruitment maneuvers and/or increased PEEP are permitted as a rescue strategy at the anesthesiologist’s discretion (Table 2). Data on deviations from the protocol (including the number of completed recruitment maneuvers and effective intraoperative PEEP levels) will be analyzed (Fig. 2).

### Standard procedures

#### Screening and inclusion

Patients are screened for inclusion and exclusion criteria during the preoperative visit with the anesthesiologist. In the absence of exclusion criteria, patients are included after providing written, signed, informed consent.

#### Randomization

Computer-generated randomized lists will be drawn up by an independent operator before the beginning of the study, using a permuted block design. The allocation sequence is stratified by center (1:1 allocation ratio) and sequentially numbered. The allocation is implemented automatically in the electronic case report form (CleanWEB™, Telemedicine Technologies S.A.S., Boulogne-Billancourt, France). The anesthesiologist in charge will assign a patient to the intervention when the patient is in the operating room with a confirmed indication for surgery.

#### Surgery

The type of drugs used for the anesthesia, the management of the CPB and fluid and transfusion strategies are implemented according to local protocols in each recruiting center. Nonetheless, the use of peridural thoracic anesthesia is not permitted. During sternal sawing, PEEP will be temporarily set to 0 cmH2O in both groups in order to prevent unnecessary pleural opening. Before aortic declamping, de-airing maneuvers with manual balloon ventilation are performed in both groups according to local protocols, with or without the use of transesophageal echocardiography and under surgical guidance.

#### Follow up

During transport from the operating room to the ICU, ventilation is operated with a self-inflating balloon or transport ventilator. If the transport ventilator is used, respiratory parameters are set according to the allocated treatment arm. A fast-track extubation protocol, defined by extubations performed before the 6th postoperative hour, is followed in all centers. The postoperative care, including sedation drugs, analgesia, fluid management, respiratory physiotherapy and the duration of the stay in the ICU, is performed according to local protocols and at the discretion of the physician in charge. The postoperative use of curative non-invasive ventilation or nasal high-flow oxygen therapy is implemented according to local protocols in each recruiting center. “Prophylactic” use (before any type of respiratory failure) of these techniques is not permitted. New invasive mechanical ventilation will be indicated at the discretion of the ICU physician in charge. The minimal ICU length of stay is 24 h.

### Study endpoints

The primary endpoint, the proportion of PPCs, is defined as a composite endpoint taking the presence of at least one of the following items during the first 7 postoperative days into account. These PPCs have been defined, in accordance with previous or ongoing studies [20, 29, 37], as follows:

1. Mild respiratory failure: SpO2 < 90% or PaO2 < 60 mmHg after breathing ambient air for 10 min (excluding hypoventilation) and corrected with an oxygen supply of 1–3 L/min with a nasal cannula
2. Moderate respiratory failure: SpO2 < 90% or PaO2 < 60 mmHg despite a 3 L/min oxygen supply with a nasal cannula (excluding hypoventilation) and corrected with an oxygen supply from 4 to 10 L/min with a face mask.
3. Severe respiratory failure: SpO2 < 90% or PaO2 < 60 mmHg despite a 10 L/min oxygen supply with a face mask (excluding hypoventilation) and corrected with an oxygen supply > 10 L/min with a high-flow face mask or with non-invasive ventilation or with high-flow nasal oxygen therapy or with invasive mechanical ventilation
4. Fast-track extubation failure associated with hypoxemia: delayed extubation after the first 6 h postoperative, associated with PaO2/FiO2 < 300
5. New invasive mechanical ventilation associated with hypoxemia, defined as PaO2/FiO2 < 300
6. Bronchospasm: new wheezing, indicating bronchodilator treatment (except preoperative chronic obstructive pulmonary disease (COPD) or asthma)
7. Severe tracheobronchial congestion: audible ronchi associated with disturbance in respiratory mechanics
8. Post-extubation respiratory acidosis defined by pH ≤ 7.30 and PaCO2 > 45 mmHg
9. Suspected pneumonia: new pulmonary infiltrate on a chest x-ray, plus at least two of the following: temperature > 38.5 °C or < 35.5 °C, leukocytosis or leukopenia (white blood cells > 12,000 cells/mm^3^ or < 4000 cells/mm^3^), purulent secretions and antibiotic treatment
10. Confirmed pneumonia: new pulmonary infiltrate on a chest x-ray plus microbiological documentation (> 10^7^ CFU/mm^3^ on expectorated sputum, > 10^5^ CFU/mm^3^ on trans-tracheal aspiration or > 10^4^ CFU/mm^3^ on bronchoalveolar lavage)
11. Pleural effusion with need for further postoperative pleural drainage
12. Radiological atelectasis: new lung opacity on a chest x-ray with a shift in the mediastinum or ipsilateral hemi-diaphragm
13. Acute respiratory distress syndrome (ARDS) as defined by the Berlin definition [38].

The secondary clinical endpoints include:

1. Each preceding PPC by postoperative day 7 analyzed individually
2. Use of non-invasive ventilation by postoperative day 7
3. Use of high-flown nasal oxygen therapy by postoperative day 7
4. Use of new invasive mechanical ventilation by postoperative day 7
5. Postoperative extrapulmonary complications analyzed individually by postoperative day 7

- Systemic inflammatory response syndrome, sepsis and septic shock (as defined in [39])
- Postoperative wound infection (sepsis with purulent wound drainage and antibiotic administration)
- Postoperative pericardial tamponade (need for re-intervention)
- De novo postoperative atrial fibrillation
- Cardiogenic pulmonary edema (acute hypoxemia with diffuse bilateral pulmonary infiltrate on a chest x-ray, high left atrial pressure on cardiac ultrasound or pulmonary capillary wedged pressure > 18 mmHg)
- Acute kidney injury (Kidney Disease: Improving Global Outcomes (KDIGO) stage 2 or 3)
- Delirium (disturbed state of consciousness and cognitive dysfunction with or without agitation)

6. Adverse events by postoperative day 7:

- postoperative pneumothorax (need for further postoperative pleural drainage)
- use of intraoperative or postoperative vasoactive drugs (excluding ephedrine and phenylephrine)
- use of high-dose inotropes (> 8 μg∙kg^− 1^∙min^− 1^ of dobutamine or > 0.8 μg∙kg^− 1^∙min^− 1^ of milrinone)
- acute postoperative bleeding with need for re-intervention before the 12th postoperative hour

7. Survival in terms of ICU-free days by postoperative day 7
8. Global mortality by postoperative day 7

### Data collection

Study data are managed with a password-protected electronic case report (CleanWEB™ operated by Telemedicine Technologies S.A.S., Boulogne-Billancourt, France).

#### Baseline data

The following baseline data are collected after the patient’s inclusion: sex, age, height, weight, BMI, American Society of Anesthesiologists (ASA) score, Euroscore II, smoking status, alcohol status, history of COPD or asthma with chronic inhalation therapy, lower respiratory tract infection in the past 3 months, abnormal preoperative chest x-ray, nutritional depletion (10% weight loss in the past 6 months), cardiovascular status (diabetes mellitus, arterial hypertension, preoperative atrial fibrillation, left ventricular ejection fraction, echocardiographic right ventricular distention defined by a right ventricle/left ventricle ratio > 1, history of stroke) and preoperative creatininemia > 200 μmol/L.

#### Intraoperative variables

During the surgery, the anesthesiologist in charge collects the following variables: type of surgery (coronary artery bypass graft, valve surgery, aortic surgery, mixed or complex surgery), need for mammary artery harvesting (unilateral or bilateral), CPB duration, aortic cross clamp duration, cardioplegia volume, intraoperative fluid volume including CPB priming (crystalloid and colloid), use of blood transfusions, need for intraoperative vasopressor (other than phenylephrine or ephedrine), need for inotropes during CPB weaning, effective tidal volume (milliliters and milliliters per kilogram of ideal body weight), intraoperative lowest, highest and main PEEP (main PEEP is defined as the PEEP used most of the time during surgery, as indicated on the ventilator monitor), complete realization of each recruitment maneuver, effective ventilation during CPB, need for protocol deviation (surgical or hemodynamic), need for rescue therapy for desaturation and calculated dynamic and static respiratory compliance at the end of surgery.

#### Postoperative variables

Respiratory assessment is carried out at least 2 h after extubation if the respiratory rate is > 10 cycles per minute. Then, patients are visited, twice a day, every postoperative day until postoperative day 7 in order to assess the presence of PPCs or secondary endpoints. Need for supplemental oxygen is assessed at every visit by measuring SpO2 and/or PaO2 after 10 min breathing room air. During the ICU stay, a daily chest x-ray is prescribed. In the surgical ward, a chest x-ray is prescribed at the discretion of the physician in charge. In the case of new or continued invasive mechanical ventilation, blood gas analysis is prescribed every 8 h in order to assess the PaO2/FiO2 ratio. In extubated patients, an arterial blood gas analysis is prescribed once a day during the ICU stay, and in the case of desaturation in the surgical ward. Echocardiography can be implemented at the discretion of the physician in charge of patient care, to diagnose cardiogenic pulmonary edema (Fig. 2).

### Sample size and power

The sample size was determined to obtain 80% power to detect a 10-point difference between the two groups in the occurrence of PPCs at day 7 (25% in the control strategy group vs 15% in the experimental strategy group). This difference is based on previous reports [1, 3, 10] and has been considered to be clinically significant. With the threshold for statistical significance set at a *P* value of 0.05, these calculations showed that 494 patients are needed (247 per group, Fig. 1). As patients will be allocated in the operating room after confirmation of the indication for surgery and followed during the 7 postoperative days in the ICU and surgical ward, a very low dropout rate is expected.

### Statistical analysis

The data will be analyzed using SPSS version 17.0 software. Patients who have at least one of the following conditions will be not included in the final analysis: patients inappropriately included despite providing consent, and patients who remove their consent. The primary analysis will be carried out according to the intention-to-treat principle. The full analysis population (including all subjects who will be randomized and will be at least evaluated at baseline) will be used in the primary analysis. No interim analysis is planned. A flow chart will be provided. The normality of the parameters will be estimated using frequency histograms and the Shapiro test. The baseline and intraoperative parameters will be described per group (“control” and “experimental”) in accordance with the Consolidated Standards of Reporting Trials (CONSORT) guidelines [40]. The proportion of PPCs at 7 postoperative days will be calculated and compared between the two groups (control and experimental) using the chi-square test or Fisher’s exact test for categorical variables (primary analysis). Multivariate analysis (secondary analysis) using logistic regression models will be performed to determine variables potentially linked to the occurrence of PPCs. Variables relevant to the models will be selected based on their clinical significance and/or a threshold *P* value ≤0.1 in the univariate analysis. The final models will estimate the odds ratios and 95% confidence intervals. The proportions of each secondary endpoint (each postoperative pulmonary complication, non-pulmonary complication, use of new invasive and non-invasive ventilation, use of high-flow nasal oxygen therapy, adverse event) will be compared between the groups. Multiple comparison corrections will be performed for non-independent outcomes. ICU-free days will be compared between the two groups. A potential center effect will be assessed by mixed effects modeling using generalized linear mixed model (SAS software, 9.4 version, GLIMMIX procedure; center as a random effect); the result will be presented as the odd ratio and its 95% CI. All of the tests will be two-tailed with a 5% significance level.

### Regulatory issues

An ethics committee approved this study (Comité de Protection de Personnes Sud Mediterranee I) on 29 February 2016 (ID RCB 2016-A00352–49). The study was registered on ClinicalTrials.gov on 15 August 2016 (NCT02866578). All eligible patients will be included in the study after obtaining signed, informed consent. At any time and for any reason, the patient can withdraw his consent. Investigators are able to terminate the study prematurely in a patient’s best interest. Should the study be discontinued, the reason will be documented on the electronic case report form. Patient data are collected anonymously on the electronic platform, as an identification number designates them. All severe adverse events are documented in the electronic case report form and declared to the Comité de Pharmacovigilance Assistance Publique des Hôpitaux de Marseille. Patient data and safety are monitored by a monitoring referent (Samir Benkouiten) and a monitoring committee (Marc Leone, Nicolas Bruder, Pascal Auquier). Samir Benkouiten will conduct monitoring visits independently. Full access to the final data set will be reserved for the main investigator (DL) and the statistician (KB) under the control of the monitoring committee.

## Discussion

Despite recent technological progress, cardiac surgery with CPB remains responsible for a high rate of respiratory morbidity [1]. This specificity results from a “two-hit” lung injury [10]. A specific pulmonary inflammation and ischemia-reperfusion injury is associated with the usual adverse effects of general anesthesia and invasive mechanical ventilation [5]. Protecting the lung during general anesthesia with specific ventilator settings has already been described. For example, the use of low tidal volumes (6–8 mL/kg of ideal body weight) has been well-validated in different types of surgery [23]. However, the use of low tidal volumes may be responsible for the development of atelectasis, particularly in the dependent region of the lung [16]. The open-lung ventilatory approach is based on systematic alveolar recruitment in order to prevent atelectrauma and increase pulmonary compliance. In parallel, the use of high levels of PEEP is necessary for maintaining this benefit [24]. Continuing to ventilate the lung during CPB, despite the absence of perfusion, can reasonably be integrated into the open-lung approach in order to prevent the formation of atelectasis during this surgical step [14]. In abdominal surgery, the benefit of the open-lung approach, in terms of PPC prevention, has not yet been proved [28, 29]. Moreover, the use of high levels of PEEP and/or lung ventilation during CPB may interact with the surgical technique, adding complexity to the surgical procedure and reducing surgical comfort. The fact that low PEEP ventilation and ventilation cessation during CPB make the surgical procedure easier has been claimed by a vast majority of cardiac surgeons. This observation may explain current practices in mechanical ventilation in cardiac surgery operating rooms [31]. Finally, the hemodynamic impact of open-lung ventilation could be more challenging for cardiac anesthesiologists. Because of its pathophysiological specificities, cardiac surgery involves a high incidence of PPCs and might particularly benefit from the open-lung approach [25, 26, 32]. However, because of a real surgical concern, the use of the open-lung approach in these patients needs to be supported by the highest level of clinical evidence that will guide the anesthesiologists and cardiac surgeons in managing ventilator settings before, during and after the CPB. The PROVECS trial is the first multicenter, randomized, controlled trial to evaluate the effect of a perioperative and multimodal ventilatory strategy depending on the open-lung approach in cardiac surgery with CPB. For feasibility concerns, specific surgical protocol deviations have been designed. The objective is thus to compare an experimental, surgeon-controlled, open-lung strategy with a conventional, low PEEP and “surgeon-friendly” approach as the control strategy.

In the experimental arm, we have chosen a multimodal approach, from intubation to extubation, in order to maximize the potential benefit effect of alveolar recruitment. Indeed, the risk of atelectasis persists during the entire mechanical ventilation period. The pressure level for recruitment maneuvers is relatively low (30 cmH2O) for hemodynamic reasons. However, by timing two recruitment maneuvers under CPB, we ensure good hemodynamic tolerance of these maneuvers. Moreover, this level of pressure prevents unintended lung harm with regard to the risk of higher transpulmonary pressures when the thorax and/or pleura are opened. The PEEP levels in each group have been empirically defined. We did not choose an individualized PEEP titration protocol because of the absence of a validated reference titration protocol and because of the high risk of hemodynamic intolerance and barotrauma [27]. In the open-lung group, the basal PEEP level of 8 cmH2O is moderate in comparison with previous studies in non-cardiac surgery [28, 29]. This will reduce the risk of lung overdistension and higher driving pressures [41]. Nonetheless, this starting level of PEEP is high enough to both prevent atelectrauma and be significantly different from the control group’s level (2cmH2O) considering the probable surgical protocol deviation. On the other hand, in the control group, the conventional ventilatory protocol corresponds in many ways to recent reports of current practices in mechanical ventilation in cardiac surgery [31] and makes possible optimal surgical comfort. During CPB, the use of ultraprotective ventilation with very low tidal volumes has been selected because of the theoretical advantages of this approach shown in previous studies of postoperative shunt fraction and inflammatory response [32]. Setting FiO2 at 40% during CPB has been planned in order to prevent absorption atelectasis secondary to lung denitrogenation while maintaining lung oxygenation by direct diffusion of alveolar oxygen [36].

The experimental arm design includes standardized protocol deviations on surgical demand. This is a crucial point. The strict application of the open-lung strategy in cardiac surgery is unrealistic and could lead to surgical complications for participating subjects. The objective of this trial is to evaluate the impact of maximizing alveolar recruitment in an intention-to-treat way. Full collaboration is therefore necessary between the anesthesiologist and the surgeon in order to adapt ventilator settings and ensure acceptable surgical comfort. The effective intraoperative settings will be registered and analyzed, and the results will be interpreted to determine the effective differences between the two groups. In this regard, we think that a per protocol analysis is not necessary because of its clinical irrelevance. The study population corresponds to daily elective surgical cases. We exclude emergent or redux surgery, and patients with severe preoperative cardiac disease because of the high risk of confounding factors in PPC assessment. The risk of hemodynamic intolerance and the complexity of the surgical procedures in these cases may lead to major protocol deviation, thus diminishing the relevance of the trial.

We opted for a binary collapsed composite of single PPCs that have a real clinical meaning in daily practice. With a consensual and unambiguous definition of PPCs, we facilitate the assessment of the primary outcome, prevent the risk of wrong diagnoses and allow for comparisons with previous or ongoing studies [20, 28, 29, 37]. For example, the “respiratory failure” outcome, based on hypoxemia evaluated with SpO2 tolerance to room air ventilation, has previously been described in different trials interested in PPCs. This is a very pragmatic definition, clinically relevant for physicians caring for patients undergoing cardiac surgery. As the relevance of the primary endpoint depends on its definition, we insist on external validity and the objective way of diagnosing each PPC used in the composite endpoint of this trial. We have chosen to exclude pneumothorax and to evaluate it as an adverse event because high PEEP may increase the incidence of pneumothorax (barotrauma or surgical trauma). Cardiogenic pulmonary edema is considered to be an extrapulmonary complication because it may bias the primary outcome regarding the potential high incidence of this event in patients with chronic heart diseases. We insist on strict confirmation of cardiogenic edema with high left atrial pressure estimated with echocardiography or a pulmonary artery catheter. Nonetheless, pneumothorax and cardiogenic pulmonary edema might lead to the primary outcome discovering whether or not they lead to hypoxemia. The need for non-invasive ventilation or high-flow nasal oxygen therapy has not been included in the primary outcome because of the absence of defined indications in the standard procedures of the trial. Curative non-invasive ventilation or high-flow nasal oxygen therapy are used depending on local protocols or the physician’s discretion. Therefore, it will be recorded as a secondary outcome. By not allowing prophylactic use (in the absence of hypoxemia) of these techniques, we avoid a potential interaction with the primary outcome. Finally, we will evaluate postoperative extra-pulmonary complications because PPCs may be related to other organ failures, such as sepsis, postoperative atrial fibrillation or acute kidney injury. Evaluating different surgical complications (such as acute postoperative bleeding with a need for re-intervention, pericardial tamponade, wound infection, or the need for high doses of inotropes) will give a safety point of view on the surgical impact of the open-lung approach.

In conclusion, the PROVECS multicenter, randomized, controlled trial aims to evaluate the impact of an open-lung multimodal and perioperative ventilatory approach on the incidence of PPCs after on-pump cardiac surgery. The strategy evaluated is optimized with regards to patient safety and surgical comfort in order to be clinically relevant. The pragmatic design of this trial will ensure that the results have a strong impact on the clinical practice of cardiac anesthesiologists and cardiac surgeons.

### Trial status

The PROVECS trial is currently recruiting patients.

## Additional files

Additional file 1: PROVECS study protocol (most recent version). (DOCX 330 kb)

Additional file 2: Standard Protocol Items: Recommendations for Interventional Trials (SPIRIT) 2013 checklist. (DOC 120 kb)

## Abbreviations

ARDS: Acute respiratory distress syndrome
BMI: Body mass index
CFU: Colony-forming unit
COPD: Chronic obstructive pulmonary disease
CPAP: Continuous positive airway pressure
CPB: Cardiopulmonary bypass
eCRF: Electronic case report form
FiO2: Fraction of inspired oxygen
HFNO: High-flow nasal oxygen therapy
I:E: Inspiratory time to expiratory time ratio
ICU: Intensive care unit
IMV: Intensive mechanical ventilation
NIV: Non-invasive ventilation
PaO2: Partial arterial pressure of oxygen
PBW: Predicted body weight
PEEP: Positive end-expiratory pressure
POD: Postoperative day
PPCs: Postoperative pulmonary complications
SpO2: Pulse oximetry
