# Supplementary material for: A perioperative surgeon-controlled open-lung approach versus conventional protective ventilation with low positive end-expiratory pressure in cardiac surgery with cardiopulmonary bypass (PROVECS): study protocol for a randomized controlled trial
Source: Trials. 2018 Nov 13;19:624. doi: 10.1186/s13063-018-2967-y (PMC6234562; doi:10.1186/s13063-018-2967-y)
Supplement: Supplementary file 1 — PROVECS study protocol (most recent version). (DOCX 330 kb) [file 13063_2018_2967_MOESM1_ESM.docx]

**RESEARCH PROTOCOL**

perioperative open lung PROtective VEntilation during Cardiac Surgery with cardio-pulmonary bypass : the PROVECS randomized controlled trial


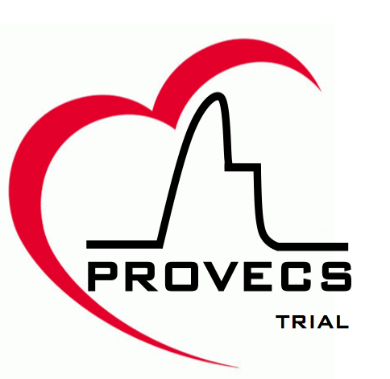


**PRINCIPAL INVESTIGATOR**

Docteur David LAGIER

Département Anesthésie Réanimation

Hôpital de la TIMONE ADULTE

Email : [david.lagier@ap-hm.fr](mailto:davdi.lagier@ap-hm.fr) Tél : 04 91 38 99 89.

**SPONSOR**

Assistance Publique-Hôpitaux de Marseille

Direction de la Recherche Clinique et de l’Innovation

80, rue Brochier

13384 Marseille CEDEX 05

Email : drci@ap-hm.fr Tél : 04 91 38 27 47.

N°IDRCB : 2016-A00352-49

Version n° : 5 du 17/01/2018


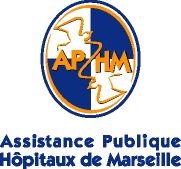


# BACKGROUND

## Postoperative Pulmonary Complications and Cardiac Surgery:

General anesthesia and mechanical ventilation impair respiratory function. Diaphragmatic hypotonia, decreased functional residual capacity (FRC), ventilator-induced lung injury (VILI)^1^, and surgical manipulation all contribute to the development of pulmonary lesions that disrupt postoperative hematosis^2,3^. Under the term "postoperative pulmonary complications" (PPCs) is grouped all the respiratory events complicating the postoperative phase^4^. PPCs, well-known in perioperative medicine, range from simple transient hypoxemia to life-threatening acute respiratory distress syndrome (ARDS). PPCs have a major impact on morbidity and mortality and mobilize invasive and expensive care resources (mechanical ventilation, intensive care). Cardiac surgery with cardiopulmonary bypass (CPB) is particularly prone to PPCs^5,6^ as compared with other less invasive surgeries. In the cardiac surgery context, postoperative hospitalization in intensive care is the rule^7^. FRC, for example, is reduced by 40 to 50% after cardiac surgery versus 20 to 30% after standard general anesthesia^8^. This feature has been known for a long time^9^ by anesthetists and cardiac surgeons. Post-CPB PPCs include atelectasis (16-88%)^10^, pleural effusion (27-95%), postoperative hypoxemia (10%)^11,12^, or ARDS (0.5%-1.7%)^13^. They are responsible for prolonging mechanical ventilation times and stay in intensive care^14^. Finally, 40% of ICU readmissions after cardiac surgery are secondary to respiratory failure^15^.

This specificity related to on-pump cardiac surgery is explained by a singular physiopathology^16,17^:

### Inflammation

During on-pump cardiac surgery, a major activation of the inflammatory cascade is found at the systemic level^18^ and, more specifically, at the pulmonary level^19,20^. Thus, massive release of cytokines, activation of complement^21^, and microcirculatory involvement by endothelial and leukocyte activation are part of the physiopathology involved during and after the intervention. This inflammatory response is secondary to surgical trauma (median sternotomy, hervest of the internal mammary artery) but also to the CPB itself (biocompatibility of the surface of the extracorporeal circuit and oxygenator, loss of pulsatility). The lung is particularly sensitive to the pro-inflammatory effects of CPB and presents several injuries predisposing to PPCs: impairment of the capillary-alveolar-capillary barrier11, impairment of pulmonary microvasculature, obstruction of small airways and mucociliary disturbances^22^.

### Ischemia-reperfusion

Moreover, aortic cross-clamping and cardioplegia are responsible for cardiac and pulmonary ischemia-reperfusion^23^ triggering oxidative stress and inflammation at the systemic and local levels. Indeed, the supply of oxygen to the lungs during a total CPB is only provided by the bronchial circulation (complete discharge of the pulmonary artery)^24,25^. We know that the blood flow in the bronchial arteries is clearly disrupted in animal models of CPB (10% of preoperative blood flow)^26^. In contrast, the impact of ventilation on bronchial circulation and bronchopulmonary anastomosis remains poorly described. It seems, however, ventilation interruption during short CPB does not cause pulmonary hypoxia^27,28^.

### Other factors

The frequent use of transfusions during cardiac surgery may contribute to the appearance of PPCs (Transfusion Related Acute Lung Injury)^29^. Diaphragmatic paralysis by iatrogenic injury to the phrenic nerve, with an estimated incidence of 5%, can also contribute to atelectasis formation^30^. Overall, the negative impact of general anesthesia and mechanical ventilation is increased by the inflammatory environment induced specifically by cardiac surgery. This “Two-hit” lung injury explains the high incidence of PPCs after on-pump cardiac surgery.

## Concept of protective ventilation

Invasive mechanical ventilation may cause mechanotrauma (volo, baro, atelectrauma) and biotrauma (inflammation) that leads to VILI associating atelectasis and alveolar overdistensions^31^. To prevent pathophysiological disturbances induced by mechanical ventilation, the concept of protective ventilation was initially developed for the management of the most severeform of lung injury: ARDS. Protective ventilation is based on the prevention of volo and barotrauma by the application of small tidal volumes (5-6 mL / kg of predicted body weight or PBW) and the respect of low plateau pressures (less than 30cmH2O) and on the prevention of atelectrauma^32^ by combining recruitment maneuvers (MR) and a high level of positive end-expiratory pressure (PEEP)^33^. The impact of protective ventilation on severe respiratory failure requiring mechanical ventilation in intensive care is clearly recognized^34^. One of the suspected protective mechanisms is notably the significant decrease in the production of inflammatory mediators (biotrauma) during the application of protective ventilation^35,36^. Thereafter, use of protective ventilation on healthy lungs in order to prevent lung injury, induced by general anesthesia, mechanical ventilation and / or surgery, is a concept that has recently aroused great interest. Indeed, the anti-inflammatory effect and the preventive effect on PPCs have been demonstrated during the application of intraoperative protective ventilation, mainly in visceral^38^ and thoracic surgery^39^. In particular, a series of clinical trials has highlighted the importance of using low tidal volumes (6-8 ml / kg PBW) to prevent PPCs^37^. On the other hand, the interest of open-lung approach (recruitment maneuvers and high level of PEEP) in the mechanical ventilation of the healthy lung is still not demonstrated as clinical studies in non-cardiac surgery shows contradictory results^37,40^.

## Protective Ventilation in Cardiac Surgery: Summary of Evidence

The effectiveness of a multimodal protective ventilation strategy in the context of on-pump cardiac surgery could be significant, but the clinical impact on the occurrence of PPCs has never been evaluated^41^. Using low tidal volume is consensual in any setting. Other protective ventilation modalities, particularly open-lung approach and continuation of ventilation during CPB^42^, have never been studied in a large clinical trial with objective clinical endpoints^43-48^. However, open-lung ventilation has been shown to decrease the inflammatory response^19^ and improve FRC^49^ following cardiac surgery. Concerning ventilation maintaining during cardiopulmonary bypass, strategies used by anesthesiologists are particularly heterogeneous. Different studies, comparing different ventilatory approaches, have shown a short-term interest regarding postoperative oxygenation parameters without showing a real clinical interest^50,51^. No clinical studies associating ventilation during CPB with pre- and post-CPB alveolar recruitment have ever been published^52,53^. Therefore, any high-level evidence support the use of an open-lung approach in cardiac surgery^54^.

## Specificities of Mechanical Ventilation during Cardiac Surgery

### Technical specificities

Cardiac surgery with CPB requires general anesthesia with invasive mechanical ventilation. After performing a median sternotomy, a mediastinal dissection step is required for placement of CPB cannulas and / or internal mammary artery graft dissection. This dissection phase is performed near the lungs with or without opening the mediastinal edge of the pleura. Pulmonary movements are a technical barrier for the heart surgeon. During the CPB, gas exchanges are fully managed by the extra-corporeal oxygenator. Mechanical ventilation can therefore be interrupted or continued provided that it does not technically disrupt the surgical procedure. At the end of the CPB, de airing maneuvers are necessary before aortic declamping. They involve effective ventilation of both lungs to drive air out of the pulmonary circulation. Ventilatory strategy in cardiac surgery has imperatives and influences the surgical approach. Thus, good cooperation must be implemented between anesthesiologists and surgeons. Given the technical impact ofventilation strategy during cardiac surgery, it appears essential to obtain solid evidence. The prevention of PPCs and surgical comfort will be the main axes for the determination of this strategy.

### Physiological specificities

Invasive mechanical ventilation interacts with cardiac function primarily through an increase in right ventricular afterload^55,56^. MR is also accompanied by a clear decrease in venous return and stroke volume. During cardiac surgery, the impact of positive pressure ventilation on cardiac output appears to be moderate with PEEP levels below 15 cmH2O^57,58.^ In addition, atelectasis treatment by the open-lung ventilation approach ultimately improves post-load of the right ventricle by reducing hypoxic vasoconstriction^59^. Cardiopulmonary interactions are therefore crucial in the adoption of a ventilatory strategy, especially in cardiopathic patients. The prevention of hypoxemia and atelectasis must be weighed against the undesirable hemodynamic effects of positive pressure.

In total, multimodal protective ventilation using the principles of open-lung ventilation could be of interest in the prevention of PPCs, particularly during on pump cardiac surgery with CPB. Strong scientific arguments are needed given the technical and hemodynamic issues associated with mechanical ventilation. This reflection justifies the objective of our study.

# OBJECTIVES

## ASSUMPTION

We hypothesize that a multimodal, perioperative and surgeon-controlled protective ventilation strategy based on open-lung approach could decrease the incidence of PPCs after on-pump cardiac surgery.

## MAIN OBJECTIVE

To determine whether a protective ventilation strategy based on open-lung approach decreases the occurrence of PPCs (all grades of severity) by postoperative day 7 after cardiac surgery with CPB.

## SECONDARY OBJECTIVES

To determine whether a protective ventilation strategy based on open-lung approach decreases the occurrence of each CRP taken independently by postoperative day 7 after cardiac surgery with CPB.

To determine whether a multimodal protective ventilation strategy based on open-lung ventilation shortens the length of stay in intensive care / intensive care in a fast-track surgery protocol.

To determine whether a multimodal protective ventilation strategy based on open-lung ventilation decreases severe postoperative extra-respiratory complications by postoperative day 7 after cardiac surgery with CPB.

# METHODS

## DESIGN OF THE STUDY

This is a multicenter, French, randomized, controlled and double-blind study of patients scheduled for cardiac surgery with CPB. Two groups will be compared: 1) Experimental strategy: surgeon-controlled open-lung ventilation; 2) Control strategy: conventional protective ventilation with low PEEP.

The recruiting will be performed in 6 French adult cardiac surgery departments.

## STUDY POPULATION

### Inclusion criteria

• Patient scheduled for cardiac surgery under CPB with median sternotomy.

### Exclusion criteria

• Age <18 years old.

• Urgent surgery: cardiac transplantation, active endocarditis, type A aortic dissection.

• Aortic arch surgery with hypothermic circulatory arrest.

• Surgery for implantation of long-term circulatory assistance.

• Redux surgery.

• Mechanical ventilation within 7 days preoperatively.

• Acute or chronic hypoxemia preoperatively with PaO2 <65 mmHg or SpO2 <95% in ambient air.

• Left ventricular systolic dysfunction with LVEF <40%.

• Severe right cardiac failure and / or major dilation of the right ventricle on preoperative echocardiography.

• Pulmonary arterial hypertension with PAPs> 50 mmHg.

• Severe chronic renal insufficiency with GFR <30 mL / min.

• Hemodynamic instability (requiring aminergic support) or preoperative shock.

• Sleep apnea syndrome.

• BMI> 35 kg / m2.

• Absence of consent.

• Patient already included in a study.

### Justification of the choice of selection criteria

Our selection criteria were defined in order to extrapolate the results to the largest number of patients. The objective is to represent the most frequently encountered population in cardiac surgery. The exclusion of patients at major risk of prolonged mechanical ventilation for respiratory (pre-existing respiratory failure) or extra-respiratory (preoperative left ventricular failure, pulmonary arterial hypertension, severe chronic renal failure) aims to exclude patients for whom the impact an intraoperative preventive strategy would be minimal with respect to the risks associated with other comorbidities (confusion bias). For the same reasons, complex or urgent surgeries with preoperative instability will not be included. We will exclude severe obesity and sleep apnea syndromes that may benefit from prophylactic non-invasive ventilation.

### Number of subjects needed

Assuming a 25% PPCs incidence (according to the definitions used in our study and data from the recent literature), to highlight a 40% relative reduction (15% PPCs incidence with treatment, it will be necessary to randomize 494 patients (2 groups of 247 patients) for a power of 80% and an alpha risk of 5%.

## ENDPOINTS

### Primary endpoint (Annexes 2 and 3)

The primary endpoint, proportion of PPCs, is defined as a composite endpoint taking account the presence of at least one of the following items during the first 7 postoperative days. These PPCs have been defined as follows:

1. Mild respiratory failure: SpO2 < 90% or partial pressure of arterial oxygen (PaO2) < 60 mmHg after breathing 10 minutes of ambient air (excluding hypoventilation) and corrected with oxygen supply from 1 to 3 L/min on nasal cannula.

2. Moderate respiratory failure: SpO2 < 90% or partial pressure of arterial oxygen (PaO2) < 60 mmHg despite a 3L/min oxygen supply on nasal cannula (excluding hypoventilation) and corrected with oxygen supply from 4 to 10 L/min on face mask.

3. Severe respiratory failure: SpO2 < 90% or partial pressure of arterial oxygen (PaO2) < 60 mmHg despite a 10L/min oxygen supply on face mask (excluding hypoventilation) and corrected with oxygen supply > 10 L/min on high-flow face mask or with non-invasive ventilation or with high flow nasal oxygenotherapy or with invasive mechanical ventilation.

4. Fast-track extubation failure associated with hypoxemia: delayed extubation after the first 6 postoperative hours associated with a PaO2/FiO2 < 300.

5. New invasive mechanical ventilation associated with hypoxemia defined as PaO2/FiO2 < 300.

6. Bronchospasm: new wheezing indicating a bronchodilators treatment (except pre-op COPD or asthma).

7. Severe tracheo-bronchial congestion: audible ronchi associated with disturbance in respiratory mechanics.

8. Post-extubation respiratory acidosis defined by pH ≤ 7.30 and PaCO2 > 45 mmHg.

9. Suspected pneumonia: new pulmonary infiltrate on chest x-ray plus at least two of the following: temperature > 38°5C or < 35.5°C, leukocytosis or leukopenia (white blood cells > 12,000 cells/mm3 or < 4000 cells/mm3), purulent secretions and antibiotic treatment.

10. Confirmed pneumonia: new pulmonary infiltrate on chest x-ray plus microbiological documentation (> 107 CFU/mm3 on expectorated sputum, >105 CFU/mm3 on trans-tracheal aspiration or > 104 CFU/mm3 on broncho-alveolar lavage).

11. Pleural effusion with need for a new postoperative pleural drainage.

12. Radiological atelectasis: new lung opacity on chest x-ray with shift of the mediastinum or the ipsilateral hemi diaphragm.

13. ARDS: as defined by the Berlin definition^60^.

### Secondary endpoints

The secondary clinical endpoints include:

1. Each preceeding PPC by postoperative day 7 analyzed individually.

2. Use of non-invasive ventilation by postoperative day 7.

3. Use of high-flown nasal oxygenotherapy by postoperative day 7.

4. Use of new invasive mechanical ventilation by postoperative day 7.

5. Postoperative extrapulmonary complications analyzed individually by postoperative day 7:

- Systemic inflammatory response syndrome, sepsis and septic shock,

- Postoperative wound infection (sepsis with wound purulent drainage and antibiotics),

- Postoperative pericardial tamponade (need for re intervention),

- De novo postoperative atrial fibrillation,

- Cardiogenic pulmonary edema (acute hypoxemia with diffuse bilateral pulmonary infiltrate on chest x-ray, high left atrial pressure on cardiac ultrasound or pulmonary capillary wedged pressure > 18 mmHg).

- Acute kidney injury (KDIGO stage 2 or 3),

- Delirium (disturbed state of consciousness and cognitive dysfunction with or without agitation).

6. Adverse events by postoperative day 7: postoperative pneumothorax (need for a new postoperative pleural drainage), use of intra or postoperative vasoactive drug (excluding ephedrine and phenylephrine), use of high dose inotropes (> 8 micg-1.kg-1.min-1 of dobutamine or > 0.8 micg-1.kg-1.min-1 of milrinone), postoperative acute bleeding with need for re intervention before the postoperative 12th hour.

7. Alive ICU-free days by postoperative day 7.

8. Global mortality by postoperative day 7.

### Endpoints’ choice justification

The interpretability of our study relies on the quality and external validity of the PPCs definitions. Therefore, our composite outcome is inspired by the PROVILHO^40^ study primary outcome conducted by the European Society of Anesthesiology. These consensual definitions were taken over by the same scientific society for the design of 2 other multicenter European ongoing studies (PROBESE trial and POPULAR trial). The length of stay in intensive care or intensive care will be interpretable through a harmonization of postoperative protocols in the various inclusion centers knowing that fast-track surgery protocols (early extubation, minimum stay of 48 hours and release in service from as possible) are already widespread. Finally, ventilation times for non-respiratory causes (hemodynamic, neurological ...) will not be taken into account in the grading of a CRP. We will favor the lowest PaO2 / FiO2 ratio in these situations.

## EXPERIMENTAL TIMELINE

### Screening and inclusion

The patient will be evaluated in pre-anesthesia visit by an anesthetisologist several days before the planned date of intervention. If the patient has all the criteria for inclusion, a clear oral and written information on the objectives and design of the protocol will be given by the physician. Then, the patient's free, informed, oral and written consent will be collected.

### In the operating room

Once the patient is admitted to the operating room and the surgery confirmed, he/she will be allocated to one of the 2 experimental groups. The allocation is automatically implemented in the electronic case report form (CleanWEB™, Telemedicine Technologies S.A.S., Boulogne-Billancourt, France).

### Postoperative follow-up

A fast-track extubation protocol, defined by extubation performed before the postoperative 6^th^ hour, is followed in all center. Minimal ICU length of stay is 24 hours. A chest x-ray and arterial blood gas will also be performed daily in intensive care (routine care). The arterial blood gas will be performed every 8 hours for patients with invasive mechanical ventilation, with the PaO2 / FiO2 ratio estimation. A cardiac ultrasound or a Swan-Ganz catheter will eliminate hypoxaemia secondary to haemodynamic overload. The patient will go out in surgery service, at the earliest on postoperative day 2, if the local criteria are met. In cardiac surgery, a daily assessment, with measurement of SpO2 in ambient air, will be performed by the physician in charge. The patient's study participation will end on the 7th postoperative day.

**PROVECS trial schedule during the study period.** *eCRF* electronic Case report form, *HFNO* High-flow nasal oxygenotherapy, *ICU* Intensive care unit, *IMV* Invesive mechanical ventilation, *NIV* Non-invasive ventilation, *PaO2* Arterial pressure in oxygen, *POD* Postoperative day, *SpO2* Pulse oximetry.


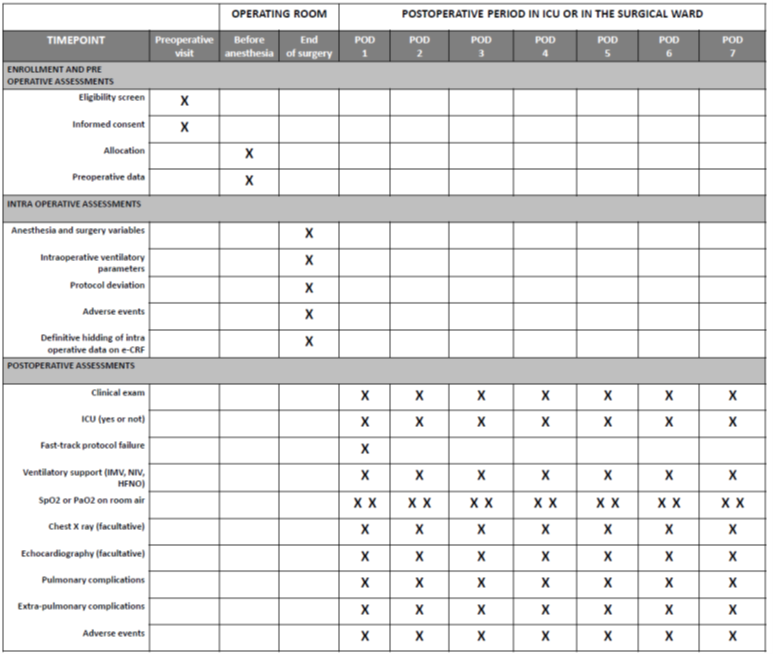


## RANDOMIZATION

Computer-generated randomized lists has been drawn up by an independent operator before the beginning of the study, using a permuted block design. The allocation sequence is stratified by center (1:1 allocation ratio) and sequentially numbered. The allocation is automatically implemented in the electronic case report form (CleanWEB™, Telemedicine Technologies S.A.S., Boulogne-Billancourt, France). The anesthesiologist in charge makes the assignation to intervention when the patient is in the operating room with a confirmed indication for surgery.

## MASKING

A double-blinding is ensured by the general anesthesia for trial participants and by the masking of outcome assessor. The irrevocable hiding of all the intraoperative datas (including ventilator settings) on the electronic case report form at the end of surgery ensure the masking of the treatment arm.

# INTERVENTIONS

## TWO-ARM VENTILATORY STRATEGIES:

Mechanical ventilation will be administered by the usual anesthetic respirators of each center. Following orotracheal intubation, controlled volume ventilation will be initiated with lowest FiO2 to provide SpO2 ≥ 94% (but at least 0.4). The tidal volume will be adjusted to 6 to 8 ml / kg of predicted weight (PBW) in both groups. The PBW will be calculated according to the following formula:

- Male: 50 + 0,91 x (height in cm - 152,4).

- Female: 45.5 + 0.91 x (height in cm - 152.4)

The respiratory rate will be adjusted to obtain normocapnia (defined as an ETCO2 between 35 and 45 mmHg). An I: E ratio of 1: 2 will be respected.

### Recruitment Maneuvers (RM)

- *Open-lung ventilation group*

In the open-lung ventilation group, a series of standardized MR will be performed. In practice, the recruitment maneuvers will be carried out by the application of a continuous airway pressure of 30 cmH2O during a timed period of 30 seconds (CPAP with the expiratory valve set at 30 cmH2O in VS or inspiratory pause with plateau pressure at 30 cmH2O).

MRs will be performed at specific times of the intervention:

1. After orotracheal intubation and insertion of the arterial catheter allowing continuous hemodynamic monitoring.

2. After CPB initiation, once the target blood flow is reached and before initiation of the ultra-protective ventilation.

3. Prior to aortic declamping following de-airing maneuvers.

4. Upon arrival in the ICU.

5. After each voluntary disconnection or not of the circuit.

- *Conventional protective ventilation group*

In the conventional protective ventilation group, no recruitment maneuver will be performed.

### Positive end-expiratory pressure (PEEP)

- *Open-lung ventilation group*

In the open-lung ventilation group, PEEP will be set at 8 cmH2O after orotracheal intubation and will be maintained until extubation in intensive care.

- *Conventional protective ventilation group*

A PEEP level of 2 cmH2O will be used from intubation to extubation.

### Ventilation during CPB

- *Open-lung ventilation group*

Ultra-protective ventilation will be implemented with a PEEP at 8 cmH2O, a tidal volume at 3 ml / kg PBW, a respiratory rate at 12 cycles per minute and a FiO2 at 0.4.

- *Conventional protective ventilation group*

The patient will be placed in spontaneous ventilation mode and a CPAP at 2 cmH2O will be used.

### Postoperative intensive care unit ventilation

The initial period of mechanical ventilation (6 hours) in intensive care will depend on the allocation of each patient. Thus, during this period, the settings of the ICU ventilator must be identical to the settings of the anesthesia ventilator, noted at the end of the procedure. An MR will be performed in the open-lung ventilation group on arrival in the intensive care unit.

## PROTOCOL DEVIATIONS

### Open-lung ventilation group (“surgeon-controlled”)

The anesthetist may deviate from the protocol at any time to ensure patient stability or at the request of the surgeon. We insist that the statistical analysis will be done in intention to treat. The level of PEEP may be decreased (by 1 cmH2O step) at any time on surgical demand for technical reasons.

The level of PEEP can be lowered (by 1 cmH2O step), on anesthesiologist decision, if one of the following complications occurs:

- Systolic blood pressure less than 90 mmHg for more than three minutes not responding to volume expansion and / or vasoconstrictor.

- Hypotension requiring a large dose of vasoactive drug.

- Severe right heart failure.

- Sustained cardiac arrhythmia.

- Hemorrhagic shock.

- Surgical complications that are life-threatening.

Any recruitment maneuver will be performed without invasive continuous monitoring of blood pressure. In case of hypotension with a systolic blood pressure lower than 80 mmHg, the MR will be immediately interrupted. The recruiting maneuvers will also be performed after surgical agreement so as not to interfere with the execution of the surgical procedure.

### Conventional protective ventilation group (“rescue strategy”)

In the conventional strategy group, in case of intraoperative hypoxemia (SpO2 < 92% despite FiO2 80%), unplanned recruitment maneuver and/or increase of PEEP level are permitted, as a rescue strategy, at the anesthesiologist discretion.

**Perioperative ventilatory protocol in each of the two treatment arm.** *CPAP* Continuous positive airway pressure, *FiO2* inspired oxygen fraction, *I:E* inspiratory time to expiratory time ratio, *PEEP* Positive end-expiratory pressure, *PBW* Predicted boby weight, *RR* Respiratory rate

|  | **Conventional Ventilation** | **Open-Lung Ventilation** |
| --- | --- | --- |
| **Ventilation before CPB** | - Tidal Volume 6-8 mL/kg PBW - PEEP 2 cmH2O - RR for ETCO2 35-45 mmHg. - Lowest FiO2 to maintain SpO2 > 94%. - I:E ratio at 1:2. | - Tidal Volume 6-8 mL/kg PBW - PEEP 8 cmH2O - RR for ETCO2 35-45 mmHg. - Lowest FiO2 to maintain SpO2 > 94%. - I:E ratio at 1:2. |
| **Systematic recruitment maneuvers** | No | Yes |
| **Ventilation during CPB** | CPAP 2 cmH2O | *Ultraprotective ventilation*   - Tidal volume 3 mL/kg PBW. - PEEP 8 cmH2O. - RR 12 cpm. - FiO2 40%. |
| **Ventilation after CPB**  (including in ICU) | - Tidal Volume 6-8 mL/kg PBW. - PEEP 2 cmH2O. - RR for ETCO2 35-45 mmHg. - Lowest FiO2 to maintain SpO2 > 94%. - I:E ratio at 1:2. | - Tidal Volume 6-8 mL/kg PBW. - PEEP 8 cmH2O. - RR for ETCO2 35-45 mmHg. - Lowest FiO2 to maintain SpO2 > 94%. - I:E ratio at 1:2. |
| **Protocol deviation** | *Rescue strategy*   - Unplanned recruitment maneuver. - +/- PEEP increase. | *Surgical or Hemodynamic deviation*   - Recruitment maneuver interruption. - PEEP decrease (1 cmH2O by 1 cmH2O step). |

# DATA PROCESSING

## ELECTRONIC CASE REPORT FORM

All data will be collected via an electronic platform on an e-CRF (electronic case report form). The platform will be developed thanks to a collaboration between the investigators and an IT service company (CleanWEB™, Telemedicine Technologies S.A.S., Boulogne-Billancourt, France), provider of the study sponsor. The goal is to optimize the collection of data by dematerializing the collection system. The computer tool will thus simplify and centralize the data retrieval, make the investigators' working time profitable and have a global and updated overview of the progress of the inclusions. The architecture of the collection sheets will be designed in collaboration with the principal investigator. The access control will be ensured by a password identification specific to each investigator.

## METHODOLOGICAL MONITORING

A monitoring referent will be appointed by the sponsor. It will ensure compliance with the methodology of the previously defined protocol and will control the data collection, their documentation and registration in accordance with good clinical practice. The investigator will vouch for the authenticity of the data and will be available for regular monitoring visit by the referent. Visits to the different sites of inclusion, opening and closing of the research and at regular intervals, will be planned by the principal investigator to evaluate the following elements:

- Respect of the protocol and procedures defined for the research.

- Verification of informed consents.

- Examination of the source documents and comparison with the data reported on the e-CRF (accuracy, missing data, coherence ...).

## DATA COLLECTION

### Preoperative

• Age and gender (years, man).

• Weight, height, BMI (kg, cm, kg / m2).

• ASA score (1 to 4).

• NYHA score (1 to 4).

• Smoking (active, weaning < 6 months, never).

• alcohol consumption (more than 2 glass of wine a day) (yes / no).

• COPD with long-term treatment (yes / no).

• Asthma under treatment (yes / no).

• Recent low respiratory tract infection (<3 months) (yes / no).

• Diabetes mellitus (yes / no).

• HTA (yes / no).

• Preoperative LVEF (%).

• Ultrasound right cardiac dilation (yes / no).

• Chronic renal failure with creatininemia > 200micmol / L (yes / no).

• Weight loss > 10% in 6 months (yes / no).

• History of stroke (yes / no).

• Euroscore II (%).

• Abnormalities on chest x-ray (yes / no)

### Intraoperative

• CPB duration(min).

• Aortic cross clamp duration (min).

• Cardioplegia volume (ml).

• Type of surgery (CABG/valvular replacement or repair/aortic surgery/complex or combined surgery)

• Tidal volume (ml and ml/kg PBW).

- Highest, lowest and main PEEP (cmH2O).
- Complete realization of each recruitment maneuver (yes/no).
- Effective ventilation during CPB (yes/no).

• Internal mammary artery graft(s) (no, uni, bilateral).

• Early bleeding with need for reintervention (yes / no).

• Fluids administration including priming: crystalloids + colloids (ml).

• RBC transfusion (units).

• Plasma transfusion (units).

• Platelet transfusion (units).

• Protocol deviation (surgical/hemodynamic/no).

• Rescue strategy for desaturation (yes / no).

• Vasopressor use (yes / no).

• Inotropes use (yes / no).

### Postoperative.

• Total duration of postoperative invasive mechanical ventilation (hours).

• Extubation at H4 (yes / no).

• Postoperative reintubation (yes / no).

• Duration of first stay in intensive care unit (hours).

• Re-admission to intensive care or intensive care (yes / no).

• Indication of NAV (yes / no).

• OHND indication (yes / no).

• Radiological pneumothorax (yes / no).

• PPCs and specify:

- Mild post-extubation hypoxemia (yes / no).

- Severe tracheo-bronchial congestion (yes / no).

- Bronchospasm (yes / no).

- Moderate post-extubation hypoxemia (yes / no).

- Post-extubation respiratory acidosis (yes / no).

- Radiological atelectasis (yes / no).

- Extubation impossible at H6 or reintubation reasons with a minimum PaO2 / FiO2 ratio < 300 (yes / no).

- Severe post-extubation hypoxemia (yes / no).

- Infectious pneumopathy suspected (yes / no).

- Infected pneumopathy proven (yes / no).

- Pleural effusion requiring drainage (yes / no).

- ARDS according to the Berlin definition (yes / no).

• Extra-respiratory complications:

- SIRS (yes / no).

- Wound infection (yes / no).

- Severe sepsis or septic shock (yes / no).

- Reintervention because of severe bleeding within the 12 first postoperative hours (yes / no).

- Surgical tamponade or pericardial effusion (yes / no).

- Postoperative atrial fibrillation (yes / no).

- Severe right, left or global heart failure requiring a high dosage of inotropic (yes / no).

• Death by postoperative day 7 (yes / no).

- Alive ICU-free days by day 7 (1,2,3,4,5,6).

## STATISTICAL ANALYSIS

All analyzes will be carried out by Dr. Karine Baumstarck methodologist-statistician of the Public Health Laboratory of the Faculty of Medicine of Timone (Aix Marseille University). The data will be analyzed using SPSS version 17.0 software. The patients who present at least one of the following conditions will be not included in the final analysis: patients inappropriately included despite providing consent and patients who remove their consent. The primary analysis will be realized according to the intention-to-treat principle. The full analysis population (including all subjects who will be randomized and will be at least evaluated at baseline) will be used in the primary analysis. No interim analysis is planned. A flow chart will be provided. The normality of the parameters will be estimated using frequency histograms and the Shapiro test. The baseline and intraoperative parameters will be described per group (‘control’ and ‘experimental’) in accordance with the CONSORT guidelines. The proportion of PPCs at 7 postoperative days will be calculated and compared between the 2 groups (‘control’ and ‘experimental’) using the chi2 test or Fisher’s exact test for categorical variables (primary analysis) Multivariate analysis (secondary analysis) using logistic regression models will be performed to determine variables potentially linked to occurrence of PPCs. Variables relevant to the models will be selected based on their clinical significance and/or a threshold p-value ≤ 0.1 in the univariate analysis. The final models will estimate the odd ratios and 95% confidence intervals. The proportions of each secondary endpoints (each post-operative pulmonary complication, non-pulmonary complications, use of new invasive and non-invasive ventilation, use of high flow nasal oxygenotherapy, adverse events) will be compared between the groups. Multiple comparison corrections will be performed for non-independent outcomes. ICU-free days will compared between the two groups. All of the tests will be two-tailed with a 5% significance level.

# ETHICAL AND LEGAL ASPECTS

## STEERING COMMITTEE

This study will be overviewed by Professor Samir Jaber, Hospital Practitioner - University Professor in Anesthesiology and Intensive Care Medicine at the Montpellier University Hospital, international expert in perioperative artificial ventilation and author of numerous anesthesia publications with high level of evidence. He will validate the protocol, will follow the progress of the study and will be able to make decisions concerning the smooth running of the study and the publication strategy.

## LEGAL FRAMEWORK

The project will be conducted in accordance with the provisions of the French Public Health Code (PHC relating to interventional biomedical research (Title II of Book 1 of the first part of the Public Health Code, Article L. 1121-1 to L.1126-11). In accordance with article L.1121-1 of the PHC, the study will be carried out under the direction of the Clinical Research and Innovation Delegation of the Public Assistance of Marseille Hospitals acting as promoter. The research will be directed and monitored by the investigators from the six inclusion centers. Dr. David Lagier will be the investigator-coordinator.

In accordance with the provisions of article L. 1121-4 as drafted by Law No. 2004-806 of 9 August 2004, the research will be implemented after receiving the favorable opinion of the Competent Ethic Committee competent (CPP Sud Mediterranée) and the authorization of the French National Agency for the Safety of Medicines and Health Products (ANSM).

Information of the participants in the research will be ensured and their consent will be collected in accordance with the provisions of Articles L. 1122-1 to 1122-2. The patient will receive complete and fair information, recalling the purpose of the study, the benefits and risks expected, the progress of the study, the right of access at all times to information concerning his health, the right to ask communication of the opinion of the CPP and the authorization of the ANSM, and the faculty for the patient to refuse to participate in the research or to withdraw his consent at any time. An information leaflet (Appendix 1) containing these elements will be given to the patients eligible for the research during the pre-anesthetic consultation. As provided for in article L. 1122-1-1, patients' consent will be collected after their information, in writing or, if that is not possible, attested by an independent third party of the sponsor and the investigator. This consent may be withdrawn at any time during the conduct of the research, and without justification, by the participating subjects. Informed consent will be collected at the pre-anesthetic visit the day before the intervention. It will be materialized by a written form (Appendix 2), approved by the CPP, and co-signed by the investigator and the patient. A first copy will be given to the patient while a second copy will be kept in the medical file.

In addition to the legislative and regulatory obligations mentioned above, the investigators also undertake to work in accordance with the ethical principles applicable to medical research involving human beings contained in the declaration of Helsinki (adopted version by the 64th General Assembly, Fortaleza, October 2013) and the rules of Good Clinical Practice applying to biomedical research on medicinal products for human use defined by the decision of 24 November 2006. They constitute a set of quality requirements in the ethical and scientific domains, which must be respected during the planning, implementation, conduct, monitoring, quality control, audit, data collection, analysis and expression of results. The respect of these good clinical practices guarantees the protection of the rights, the security and the protection of the persons who are amenable to this research and the preservation of their anonymity as well as the credibility (integrity, authenticity) and the precision of the data and the results of this research. The investigators undertake to provide a copy of their personal curriculum vitae.

## INSURANCE

In accordance with articles R.1121-5 to R. 1121-10 of the PHC, an insurance from an authorized company will be underwritten by the sponsor (Public Assistance of the Hospitals of Marseille), to guarantee the promoter as well that of any intervener (doctor and staff involved) in the course of the research work.

## CONFIDENTIALITY

The computer processing of the data relating to this project will be carried out in accordance with the reference methodology validated by the French National Commission for Informatics and Freedoms (CNIL) and established by the Advisory Committee on Information Processing in the field of health research (Decision of 05/01/2006, Reference Methodology MR-001). The information collected from patients will be strictly confidential.

In practice: the anonymity of the subjects will be ensured by an inclusion number. The paper data (consent) will be kept in a locked room. The computer data will be declared to the CNIL according to the adapted procedure and access to the electronic platform for data collection will be secured by a password identification system. The data of the e-CRF will be stored on a secure server.

## AMENDMENTS TO THE PROTOCOL

Changes to the protocol will have to be qualified as substantial or not. Depending on their nature, they will be the subject of a new opinion by the Ethic comittee and / or an authorization from the competent authority.

## SAFETY ISSUES - MANAGEMENT OF ADVERSE EVENTS

### Definitions of adverse events

- Adverse event: any harmful event occurring in a person who is amenable to biomedical research whether or not this event is linked to research.

- Serious adverse event (SAE):

• event whose evolution is fatal,

• or endangering the life of the person who is suitable for research,

• or which causes a disability or significant or long-term disability,

• or that causes hospitalization or prolongation of hospitalization,

• or which results in an abnormality or congenital malformation,

• or any other event that does not meet the qualifications listed above, but may be considered "potentially serious",

• or medically relevant event as determined by the investigator,

• or an event requiring medical intervention to prevent progression to one of the above conditions.

### Investigators duty

- *Methods for detecting and collecting adverse events*

All adverse events should be researched, reported and recorded, treated and evaluated from the first visit (inclusion) to the end of the study. They will be collected at any time if the investigator is aware of it and believes that there is a causal link between the event and the research. Adverse events are collected during clinical, laboratory or other investigations by the investigator. All adverse events will be noted on the adverse event collection forms. A page will be provided for this purpose on the e-CRF.

- *Statement of the adverse event*

The investigator evaluates each adverse event in terms of its severity. He must notify the promoter, within 24 hours from the day he becomes aware. The investigator should document the event as best as possible, provide medical diagnosis where possible and establish a causal link between the event and the research protocol. The declaration is forwarded to the sponsor using the signed and dated declaration form (located on the e-CRF web platform), along with copies of laboratory results or test or test reports. These documents should remain anonymous and include the patient's code number.

The investigator must ensure that relevant follow-up information is provided to the sponsor within 8 days of the first report. The investigator should follow the patient until stabilization or until the patient returns to the previous state, even if the patient has left the trial. He must inform the promoter of the evolution of the EIG. The notification can be made by fax or email to the promoter using the duly completed EIG declaration form which can be found on the e-CFR web platform and in the investigative file and to: APHM:

80, rue Brochier, 13354 Marseille Cedex 05

Telephone: 04 91 38 27 47. Fax: 04 91 38 14 79

E-mail: drci@ap-hm.fr

### Independent monitoring committee

An independent monitoring committee will be formed. It will include two anesthesiologist professors (Pr Nicolas Bruder and Pr Marc Leone - APHM) and a statistician (Pr Pascal Auquier - APHM).

#

# REFERENCES

1. Pinhu L, Whitehead T, Evans T, Griffiths M. Ventilator-associated lung injury. *Lancet.* 2003;361(9354):332-340.

2. Rock P, Rich PB. Postoperative pulmonary complications. *Current opinion in anaesthesiology.* 2003;16(2):123-131.

3. Canet J, Gallart L, Gomar C, et al. Prediction of postoperative pulmonary complications in a population-based surgical cohort. *Anesthesiology.* 2010;113(6):1338-1350.

4. Jeong BH, Shin B, Eom JS, et al. Development of a prediction rule for estimating postoperative pulmonary complications. *PloS one.* 2014;9(12):e113656.

5. Ng CS, Wan S, Yim AP, Arifi AA. Pulmonary dysfunction after cardiac surgery. *Chest.* 2002;121(4):1269-1277.

6. Stephan F, Barrucand B, Petit P, et al. High-Flow Nasal Oxygen vs Noninvasive Positive Airway Pressure in Hypoxemic Patients After Cardiothoracic Surgery: A Randomized Clinical Trial. *Jama.* 2015;313(23):2331-2339.

7. Wynne R, Botti M. Postoperative pulmonary dysfunction in adults after cardiac surgery with cardiopulmonary bypass: clinical significance and implications for practice. *American journal of critical care : an official publication, American Association of Critical-Care Nurses.* 2004;13(5):384-393.

8. Nicholson DJ, Kowalski SE, Hamilton GA, Meyers MP, Serrette C, Duke PC. Postoperative pulmonary function in coronary artery bypass graft surgery patients undergoing early tracheal extubation: a comparison between short-term mechanical ventilation and early extubation. *Journal of cardiothoracic and vascular anesthesia.* 2002;16(1):27-31.

9. Taggart DP, el-Fiky M, Carter R, Bowman A, Wheatley DJ. Respiratory dysfunction after uncomplicated cardiopulmonary bypass. *The Annals of thoracic surgery.* 1993;56(5):1123-1128.

10. Neves FH, Carmona MJ, Auler JO, Jr., Rodrigues RR, Rouby JJ, Malbouisson LM. Cardiac compression of lung lower lobes after coronary artery bypass graft with cardiopulmonary bypass. *PloS one.* 2013;8(11):e78643.

11. Badenes R, Lozano A, Belda FJ. Postoperative pulmonary dysfunction and mechanical ventilation in cardiac surgery. *Critical care research and practice.* 2015;2015:420513.

12. Weiss YG, Merin G, Koganov E, et al. Postcardiopulmonary bypass hypoxemia: a prospective study on incidence, risk factors, and clinical significance. *Journal of cardiothoracic and vascular anesthesia.* 2000;14(5):506-513.

13. Messent M, Sullivan K, Keogh BF, Morgan CJ, Evans TW. Adult respiratory distress syndrome following cardiopulmonary bypass: incidence and prediction. *Anaesthesia.* 1992;47(3):267-268.

14. Ranucci M, Ballotta A, La Rovere MT, Castelvecchio S, Surgical, Clinical Outcome Research G. Postoperative hypoxia and length of intensive care unit stay after cardiac surgery: the underweight paradox? *PloS one.* 2014;9(4):e93992.

15. Kogan A, Cohen J, Raanani E, et al. Readmission to the intensive care unit after "fast-track" cardiac surgery: risk factors and outcomes. *The Annals of thoracic surgery.* 2003;76(2):503-507.

16. Clark SC. Lung injury after cardiopulmonary bypass. *Perfusion.* 2006;21(4):225-228.

17. Huffmyer JL, Groves DS. Pulmonary complications of cardiopulmonary bypass. *Best practice & research. Clinical anaesthesiology.* 2015;29(2):163-175.

18. Paparella D, Yau TM, Young E. Cardiopulmonary bypass induced inflammation: pathophysiology and treatment. An update. *European journal of cardio-thoracic surgery : official journal of the European Association for Cardio-thoracic Surgery.* 2002;21(2):232-244.

19. Reis Miranda D, Gommers D, Struijs A, et al. Ventilation according to the open lung concept attenuates pulmonary inflammatory response in cardiac surgery. *European journal of cardio-thoracic surgery : official journal of the European Association for Cardio-thoracic Surgery.* 2005;28(6):889-895.

20. Wrigge H, Uhlig U, Baumgarten G, et al. Mechanical ventilation strategies and inflammatory responses to cardiac surgery: a prospective randomized clinical trial. *Intensive care medicine.* 2005;31(10):1379-1387.

21. Gu YJ, Mariani MA, Boonstra PW, Grandjean JG, van Oeveren W. Complement activation in coronary artery bypass grafting patients without cardiopulmonary bypass: the role of tissue injury by surgical incision. *Chest.* 1999;116(4):892-898.

22. Sanchez-Veliz R, Carmona MJ, Otsuki DA, et al. Impact of Cardiopulmonary Bypass on Respiratory Mucociliary Function in an Experimental Porcine Model. *PloS one.* 2015;10(8):e0135564.

23. Williams WG, Manley RW, Drew C. Pulmonary circulatory arrest. *Thorax.* 1965;20(6):523-527.

24. Pearse DB, Wagner EM. Role of the bronchial circulation in ischemia-reperfusion lung injury. *Journal of applied physiology.* 1994;76(1):259-265.

25. Dodd-o JM, Welsh LE, Salazar JD, et al. Effect of bronchial artery blood flow on cardiopulmonary bypass-induced lung injury. *American journal of physiology. Heart and circulatory physiology.* 2004;286(2):H693-700.

26. Schlensak C, Doenst T, Preusser S, Wunderlich M, Kleinschmidt M, Beyersdorf F. Bronchial artery perfusion during cardiopulmonary bypass does not prevent ischemia of the lung in piglets: assessment of bronchial artery blood flow with fluorescent microspheres. *European journal of cardio-thoracic surgery : official journal of the European Association for Cardio-thoracic Surgery.* 2001;19(3):326-331; disciussion 331-322.

27. Loer SA, Kalweit G, Tarnow J. Effects of ventilation and nonventilation on pulmonary venous blood gases and markers of lung hypoxia in humans undergoing total cardiopulmonary bypass. *Critical care medicine.* 2000;28(5):1336-1340.

28. Schutte H, Hermle G, Seeger W, Grimminger F. Vascular distension and continued ventilation are protective in lung ischemia/reperfusion. *American journal of respiratory and critical care medicine.* 1998;157(1):171-177.

29. Silliman CC, Ambruso DR, Boshkov LK. Transfusion-related acute lung injury. *Blood.* 2005;105(6):2266-2273.

30. Wilcox P, Baile EM, Hards J, et al. Phrenic nerve function and its relationship to atelectasis after coronary artery bypass surgery. *Chest.* 1988;93(4):693-698.

31. Slutsky AS, Ranieri VM. Ventilator-induced lung injury. *The New England journal of medicine.* 2014;370(10):980.

32. Lachmann B. Open up the lung and keep the lung open. *Intensive care medicine.* 1992;18(6):319-321.

33. Briel M, Meade M, Mercat A, et al. Higher vs lower positive end-expiratory pressure in patients with acute lung injury and acute respiratory distress syndrome: systematic review and meta-analysis. *Jama.* 2010;303(9):865-873.

34. Ventilation with lower tidal volumes as compared with traditional tidal volumes for acute lung injury and the acute respiratory distress syndrome. The Acute Respiratory Distress Syndrome Network. *The New England journal of medicine.* 2000;342(18):1301-1308.

35. Ranieri VM, Suter PM, Tortorella C, et al. Effect of mechanical ventilation on inflammatory mediators in patients with acute respiratory distress syndrome: a randomized controlled trial. *Jama.* 1999;282(1):54-61.

36. Zupancich E, Paparella D, Turani F, et al. Mechanical ventilation affects inflammatory mediators in patients undergoing cardiopulmonary bypass for cardiac surgery: a randomized clinical trial. *The Journal of thoracic and cardiovascular surgery.* 2005;130(2):378-383.

37. Serpa Neto A, Hemmes SN, Barbas CS, et al. Protective versus Conventional Ventilation for Surgery: A Systematic Review and Individual Patient Data Meta-analysis. *Anesthesiology.* 2015.

38. Futier E, Constantin JM, Paugam-Burtz C, et al. A trial of intraoperative low-tidal-volume ventilation in abdominal surgery. *The New England journal of medicine.* 2013;369(5):428-437.

39. Michelet P, D'Journo XB, Roch A, et al. Protective ventilation influences systemic inflammation after esophagectomy: a randomized controlled study. *Anesthesiology.* 2006;105(5):911-919.

40. Anaesthesiology PNIftCTNotESo, Hemmes SN, Gama de Abreu M, Pelosi P, Schultz MJ. High versus low positive end-expiratory pressure during general anaesthesia for open abdominal surgery (PROVHILO trial): a multicentre randomised controlled trial. *Lancet.* 2014;384(9942):495-503.

41. Romagnoli S, Ricci Z. Lung protective ventilation in Cardiac Surgery. *Heart, lung and vessels.* 2015;7(1):5-6.

42. Schreiber JU, Lance MD, de Korte M, Artmann T, Aleksic I, Kranke P. The effect of different lung-protective strategies in patients during cardiopulmonary bypass: a meta-analysis and semiquantitative review of randomized trials. *Journal of cardiothoracic and vascular anesthesia.* 2012;26(3):448-454.

43. Dyhr T, Laursen N, Larsson A. Effects of lung recruitment maneuver and positive end-expiratory pressure on lung volume, respiratory mechanics and alveolar gas mixing in patients ventilated after cardiac surgery. *Acta anaesthesiologica Scandinavica.* 2002;46(6):717-725.

44. Dyhr T, Nygard E, Laursen N, Larsson A. Both lung recruitment maneuver and PEEP are needed to increase oxygenation and lung volume after cardiac surgery. *Acta anaesthesiologica Scandinavica.* 2004;48(2):187-197.

45. John LC, Ervine IM. A study assessing the potential benefit of continued ventilation during cardiopulmonary bypass. *Interactive cardiovascular and thoracic surgery.* 2008;7(1):14-17.

46. Koner O, Celebi S, Balci H, Cetin G, Karaoglu K, Cakar N. Effects of protective and conventional mechanical ventilation on pulmonary function and systemic cytokine release after cardiopulmonary bypass. *Intensive care medicine.* 2004;30(4):620-626.

47. Minkovich L, Djaiani G, Katznelson R, et al. Effects of alveolar recruitment on arterial oxygenation in patients after cardiac surgery: a prospective, randomized, controlled clinical trial. *Journal of cardiothoracic and vascular anesthesia.* 2007;21(3):375-378.

48. Murphy GS, Szokol JW, Curran RD, Votapka TV, Vender JS. Influence of a vital capacity maneuver on pulmonary gas exchange after cardiopulmonary bypass. *Journal of cardiothoracic and vascular anesthesia.* 2001;15(3):336-340.

49. Reis Miranda D, Struijs A, Koetsier P, et al. Open lung ventilation improves functional residual capacity after extubation in cardiac surgery. *Critical care medicine.* 2005;33(10):2253-2258.

50. Altmay E, Karaca P, Yurtseven N, et al. Continuous positive airway pressure does not improve lung function after cardiac surgery. *Canadian journal of anaesthesia = Journal canadien d'anesthesie.* 2006;53(9):919-925.

51. Gagnon J, Laporta D, Beique F, Langlois Y, Morin JF. Clinical relevance of ventilation during cardiopulmonary bypass in the prevention of postoperative lung dysfunction. *Perfusion.* 2010;25(4):205-210.

52. Apostolakis EE, Koletsis EN, Baikoussis NG, Siminelakis SN, Papadopoulos GS. Strategies to prevent intraoperative lung injury during cardiopulmonary bypass. *Journal of cardiothoracic surgery.* 2010;5:1.

53. Loeckinger A, Kleinsasser A, Lindner KH, Margreiter J, Keller C, Hoermann C. Continuous positive airway pressure at 10 cm H(2)O during cardiopulmonary bypass improves postoperative gas exchange. *Anesthesia and analgesia.* 2000;91(3):522-527.

54. Claxton BA, Morgan P, McKeague H, Mulpur A, Berridge J. Alveolar recruitment strategy improves arterial oxygenation after cardiopulmonary bypass. *Anaesthesia.* 2003;58(2):111-116.

55. Vieillard-Baron A, Loubieres Y, Schmitt JM, Page B, Dubourg O, Jardin F. Cyclic changes in right ventricular output impedance during mechanical ventilation. *Journal of applied physiology.* 1999;87(5):1644-1650.

56. Schmitt JM, Vieillard-Baron A, Augarde R, Prin S, Page B, Jardin F. Positive end-expiratory pressure titration in acute respiratory distress syndrome patients: impact on right ventricular outflow impedance evaluated by pulmonary artery Doppler flow velocity measurements. *Critical care medicine.* 2001;29(6):1154-1158.

57. Biondi JW, Schulman DS, Soufer R, et al. The effect of incremental positive end-expiratory pressure on right ventricular hemodynamics and ejection fraction. *Anesthesia and analgesia.* 1988;67(2):144-151.

58. Reis Miranda D, Klompe L, Mekel J, et al. Open lung ventilation does not increase right ventricular outflow impedance: An echo-Doppler study. *Critical care medicine.* 2006;34(10):2555-2560.

59. Celebi S, Koner O, Menda F, Korkut K, Suzer K, Cakar N. The pulmonary and hemodynamic effects of two different recruitment maneuvers after cardiac surgery. *Anesthesia and analgesia.* 2007;104(2):384-390.

60. Force ADT, Ranieri VM, Rubenfeld GD, et al. Acute respiratory distress syndrome: the Berlin Definition. *Jama.* 2012;307(23):2526-2533.

**APPENDIX 1:** Written information to participants

**Multimodal protective ventilation in cardiac surgery with cardiopulmonary bypass (PROVECS)**

Promoter:

Delegation to Clinical Research and Innovation (DRCI)

Public Assistance of Marseille Hospitals

Tel: 04 91 38 27 47.

Dear Madam/ Sir,

Your state of health requires the completion of cardiac surgery under cardiopulmonary bypass and general anesthesia. We invite you to participate in a clinical research study as part of this intervention. This document is intended to help you make a decision about your possible participation. A reflection period will be left until the day before the intervention. You can be assisted by someone you trust.

Cardiac surgery exposes to a risk of respiratory complications during the first postoperative days. The mechanisms of this type of complications are multiple. Among these, atelectasis (collapse of lung areas that no longer participate in gas exchange) can be formed. Their persistence after the awakening of the procedure leads to an increase in oxygen requirements that will be administered by nasal goggles or a face mask. They can facilitate the appearance of other respiratory complications (such as respiratory infection) and lead to lengthen your stay in intensive care. It is possible that the settings of the ventilator (breathing apparatus) parameters that we use during the whole phase of general anesthesia may prevent the occurrence of these atelectasis. The use of ventilatory maneuvers to open the lungs and keep them open could be effective. The interest of this method has been little studied in cardiac surgery. The objective of our study is therefore to compare this artificial ventilation strategy with that which is usually used. Patients from Marseille, Paris, Lyon, Starsbourg, Bordeaux and Montpellier University Hospitals will participate in this study. If you agree, a draw will decide whether you will benefit from a conventional artificial ventilation method or the method we wish to evaluate. Neither you nor the doctor who will examine you after the procedure will know which method was used. The anesthetist who will take care of you, however, will know it. It will ensure your anesthesia, pain management and your safety as it should. Participation in this research will not affect your surgical treatment and anesthesia management. If you agree to integrate this study, the total duration of your participation will be 7 days from the date of the surgery, which corresponds to the minimal expected length of stay in the hospital after a cardiac operation. During this time, the medical and surgical team will continue to follow you, as is usually done after this type of surgery, until you leave the hospital. You will be examined every day by an anesthesiologist-intensivist whether you participate in the study or not. In particular, it will measure oxygen saturation with a sensor placed at the tip of a finger. No additional supplementary examination (blood test, radiological examination) will be added to the usual examinations. For scientific reasons, you will not be able, during the intervention and for the next 7 days, to participate in another research protocol. Your participation in this study will not result in any special risks other than those of surgery and anesthesia. Indeed, the ventilation technique studied in this research is already widely used in intensive care for patients with severe respiratory problems who must remain connected to a respirator for several days. We know that the side effects associated with this technique (low blood pressure especially) are infrequent and very quickly reversible. If your blood pressure goes down, the ventilation technique will be immediately adapted by the anesthesiologist to normalize your condition, as in any type of surgery.

This study will be conducted according to the provisions of the Public Health Code for interventional biomedical research (articles L. 1121-1 to L.1126-11) and will respect the rules of Good Clinical Practice applicable to biomedical research defined by the decision of November 24, 2006. Your participation in this biomedical research will not generate any additional costs for you compared to those you would have in the usual follow-up of this disease. However, to participate in this research you must be affiliated or benefit from a social security scheme. The Public Assistance of Marseille Hospitals, which organizes this biomedical research as a promoter, has taken out insurance in accordance with the legal provisions. In the event that your state of health is impaired by your participation in the study, you will be entitled to receive compensation under this specific insurance contract.

This research received the favorable opinion of the Committee for the Protection of South Mediterranean People and the prior authorization of the National Agency for the Safety of Medicines and Health Products.

As part of the biomedical research, a computer processing of your personal data will be implemented to allow analysis of the results. For this purpose, your medical data will be transmitted to the Research Promoter or to persons acting on his behalf. Anonymity will be respected and your data will be identified by a code number and your initials. These data may also, under conditions ensuring their confidentiality, be transmitted to the French health authorities.

In accordance with the provisions of law relating to “data, files and freedoms” you have a right of access and rectification. You thus have a right of opposition to the transmission of the data covered by the professional secrecy and likely to be used within the framework of this research. You can also access directly or through a doctor of your choice to all of your medical data. You are free to accept or refuse to participate in this research. In addition, you can exercise your right to withdraw from this research at any time. Not participating in this research will not change the quality of care that will be provided. You can ask at any time for further explanations about the study to the care team. In addition, you may be kept informed of the overall results of this research at the end of the study. When you have read this information note and obtained the answers to your questions, you will be asked, if you agree, to give your written consent by signing a document prepared for this purpose.

**APPENDIX 2:** Consent Form:

**Multimodal protective ventilation in cardiac surgery with cardiopulmonary bypass (PROVECS)**

Consent form for participation in biomedical research

I, (undersigned) Ms, M. (full name) .........................................................................................................

Birth date..................................................................................................................................................

Address………………………………………………………………………………………………………………………………………………..

Declare:

- that the Doctor (full name) ................................................................ asked me to participate in the aforementioned study,

- that he explained to me in detail the protocol and,

- that he has, among other things, made me known:

• the purpose, method and duration of the study.

• constraints and potential risks incurred.

• my right to refuse to participate in the study.

• my right to withdraw from the study at any time without justification.

• my obligation to enroll in a social security scheme.

• that, if I wish, at the end, I will be informed by the investigating doctor of its overall results.

• that I would not be allowed to participate in other clinical trials for a period of 7 days from the date of intervention.

• that the Committee for the Protection of South-Mediterranean People has issued a favorable opinion on ....

• that the National Agency for the Safety of Medicines and Health Products has issued an authorization for this study.

• in the framework of this study the promoter, the Public Assistance of Marseille Hospitals, has taken out insurance covering this research.

Investigational information collected by the investigator will be treated confidentially. I agree that the data recorded during this research may be subject to anonymous computer processing. I have noted that the right of access provided by law relating to computers, files and freedoms is exercised at all times from the doctor who follows me in the course of research and who knows my identity. I will be able to exercise my right of rectification and opposition with the same doctor who will contact the research sponsor.

After freely discussing and obtaining answers to all my questions, I freely and voluntarily agree to participate in this biomedical research under the conditions specified in the information and consent form.

Name and first name of the patient: ..........................................

Date: ....... / ....... / .......

Signature:

Name of the investigator: .........................................

Date: ....... / ....... / .......

Signature:
